# Green synthesis of pyridine-benzofuran hybrids with anti-inflammatory activity alongside *in silico* study

**DOI:** 10.1016/j.isci.2026.117389

**Published:** 2026-09-07

**Authors:** Haniye Salehi, Valiollah Hajhashemi, Ghadamali Khodarahmi, Parvin Asadi

**Affiliations:** 1 Department of Medicinal Chemistry, School of Pharmacy and Pharmaceutical Sciences, Isfahan University of Medical Sciences, Isfahan, Iran; 2 Department of Pharmacology and Toxicology, School of Pharmacy and Pharmaceutical Sciences, Isfahan University of Medical Sciences, Isfahan, Iran; 3 Pharmaceutical Sciences Research Center, Isfahan University of Medical Sciences, Isfahan, Iran; 4 Bioinformatics Research Center, Isfahan University of Medical Science, Isfahan, Iran

**Keywords:** benzofuran, anti-inflammation, naturally occurring aldehydes, docking

## Abstract

A series of anti-inflammatory pyridine-benzofuran hybrid derivatives were synthesized from 2-acetylbenzofuran and natural aldehydes via a mild and efficient multicomponent reaction. Structures were confirmed using infrared (IR) and NMR spectroscopy. Anti-inflammatory effects were evaluated in the carrageenan-induced paw edema model in adult male rats. Among all tested compounds, **7a** and **7e** demonstrated the most potent dose-dependent inhibition, reaching 61.2% and 60.3% at 200 mg/kg (∗∗*p* < 0.001), respectively, compared with indomethacin (78.5% at 10 mg/kg). Molecular docking studies against the COX-2 receptor identified compounds **7a**, **7b**, and **7f** as having the most favorable binding energies (below -8 kcal/mol). Integrating *in vivo* and *in silico* findings, compounds **7a** and **7f** emerged as top candidates. Compound **7a** was selected for a 100-ns molecular dynamics simulation, which confirmed the stability of the ligand-protein complex and sustained effective interactions with the enzyme, establishing **7a** as a promising anti-inflammatory lead candidate.

## Highlights

• New pyridine-benzofuran hybrids were synthesized via multicomponent reaction
• Compunds **7a** and **7f** showed the best anti-inflammatory effects in adult male rats
• Compounds **7a** and **7f** demonstrated good binding energy in molecular docking study
• **7a**-protein complex was stable for a 100 ns in molecular dynamics simulation

## Graphical abstract

**Figure:**
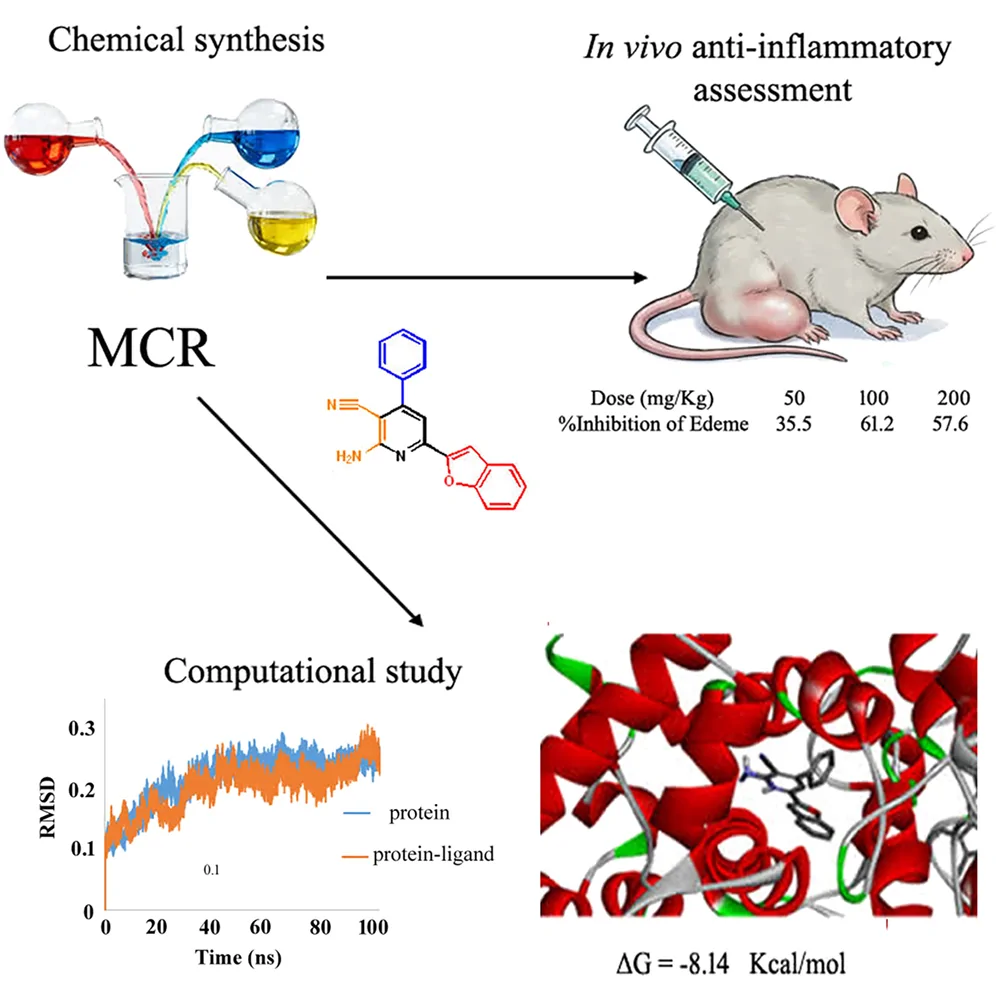

## Teaser

Spectroscopy; Natural product synthesis; Medical biochemistry

## Introduction

Inflammation is a biological response triggered by various stimuli, including physical injury, reduced blood supply (ischemic injury), infection, toxins, pathogens, and other harmful factors.^1^ Controlled inflammation is a beneficial response from living tissues aimed at restoring the structure and function of the tissue. However, if the pathogenic factor is not eliminated or the body’s regulation of inflammation is dysfunctional, it can lead to irreversible damage. Chronic inflammation paves the way for disorders such as autoimmune diseases, cancer, Alzheimer’s disease, and diabetes.^2^ Currently, the most widely used and commonly prescribed medications for the treatment of inflammation and pain are nonsteroidal anti-inflammatory drugs (NSAIDs). NSAIDs are extensively employed in the management of acute and chronic pain, musculoskeletal disorders, postoperative discomfort, rheumatoid arthritis, osteoarthritis, and various inflammatory conditions.^3^^,^^4^

The primary mechanism of NSAIDs is based on the inhibition of cyclooxygenase (COX) enzymes, achieved through interference with the arachidonic acid metabolic pathway and suppression of thromboxane and prostaglandin synthesis.^5^ Two well-characterized isoforms of the COX enzyme (COX-1 and COX-2) have distinct physiological roles. COX-1 contributes to gastric mucosal protection via prostaglandin production and plays a key role in thromboxane synthesis by platelets. In contrast, COX-2 is inducible, and its expression is upregulated in response to inflammatory stimuli such as cytokines, growth factors, and endotoxins. For the treatment of inflammation, drug development efforts shifted toward newer agents with greater selectivity for COX-2, such as celecoxib, rofecoxib, and valdecoxib.^6^ While selective COX-2 inhibitors proved effective in alleviating pain and reducing gastrointestinal damage, they were later linked to an increased risk of cardiovascular events, leading to the withdrawal of some agents from the pharmaceutical market.^7^

**Scheme 1:**
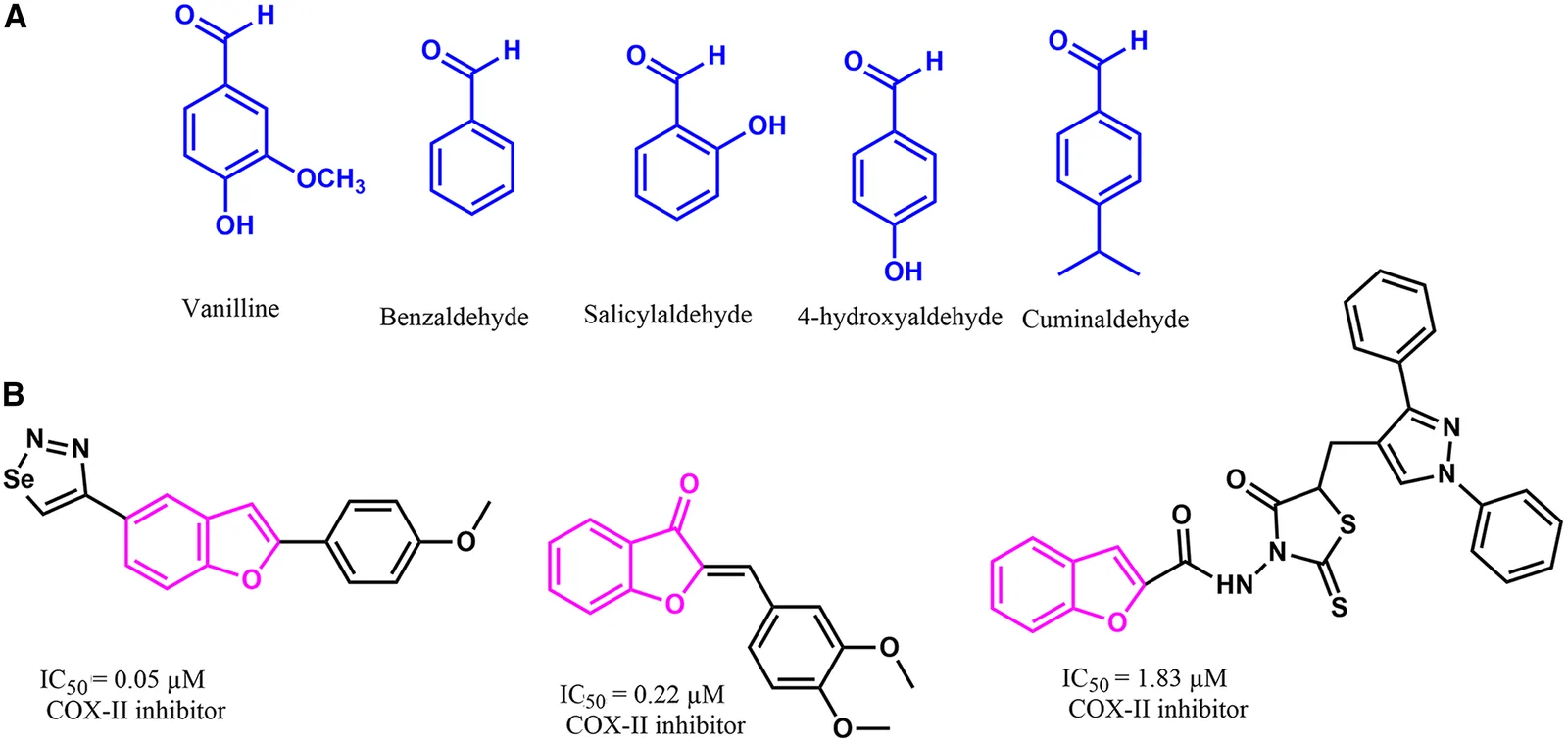
Representative naturally occurring aldehydes and benzofuran derivatives evaluated as anti-inflammatory agents (A) Structure of some naturally occurring aldehydes with anti-inflammatory activity. (B) Structure of some benzofuran derivatives with anti-inflammatory activity.

Natural products have proven to be valuable therapeutic tools for controlling inflammation, yielding very favorable results in both *in vitro* and *in vivo* studies. The hallmark features of natural compounds that make them attractive for therapy include multitargeting activity, low toxicity, and low immunogenicity (even at high doses).^8^ Among the various chemical structures of natural compounds, many aldehydes have demonstrated notable anti-inflammatory properties. The most important natural aldehydes with anti-inflammatory activity include vanillin,^9^ benzaldehyde,^10^ salicylaldehyde,^11^ anisaldehyde,^12^ cumin aldehyde,^13^ cinnamaldehyde,^14^ cannabinol aldehyde,^15^ citral,^16^ and protocatechuic aldehyde^17^ (Scheme 1). On the other hand, both natural and synthetic products containing the benzofuran ring have demonstrated a broad spectrum of biological and pharmacological activities, including antimicrobial, antioxidant, anti-inflammatory, antifungal, anti-HIV, antitumor, and antiplatelet effects.^18^^,^^19^ Consequently, they may be helpful as leads for the design and development of novel potential therapeutic agents. It has been shown that several natural benzofurans, as well as benzofuran hybrids with other heterocyclic rings, exhibit notable anti-inflammatory activity^20^^,^^21^ (Scheme 1).

The application of multicomponent reactions (MCRs) as an efficient synthetic strategy enables the rapid and targeted design of hybrid compounds with complex structures in a single step. These reactions by reducing the number of synthetic steps, improving yields, and offering structural diversity, are well aligned with the principles of green chemistry and sustainable development.^22^^,^^23^

In this project, based on the anti-inflammatory activity of benzofuran derivatives and naturally occurring aldehydes, new pyridine-benzofuran hybrid derivatives were synthesized and evaluated for their anti-inflammatory effects. The structures of the compounds were identified using infrared (IR) and NMR techniques, and subsequently their anti-inflammatory effects were evaluated in an animal model. Finally, the potential interaction of these compounds with the COX-2 receptor was examined through docking studies, and one of the best compounds, according to biological tests and docking studies, was further investigated through molecular dynamics (MD) simulation.

## Results

### Synthesis of pyridine-benzofuran hybrid derivatives

**Scheme 2:**
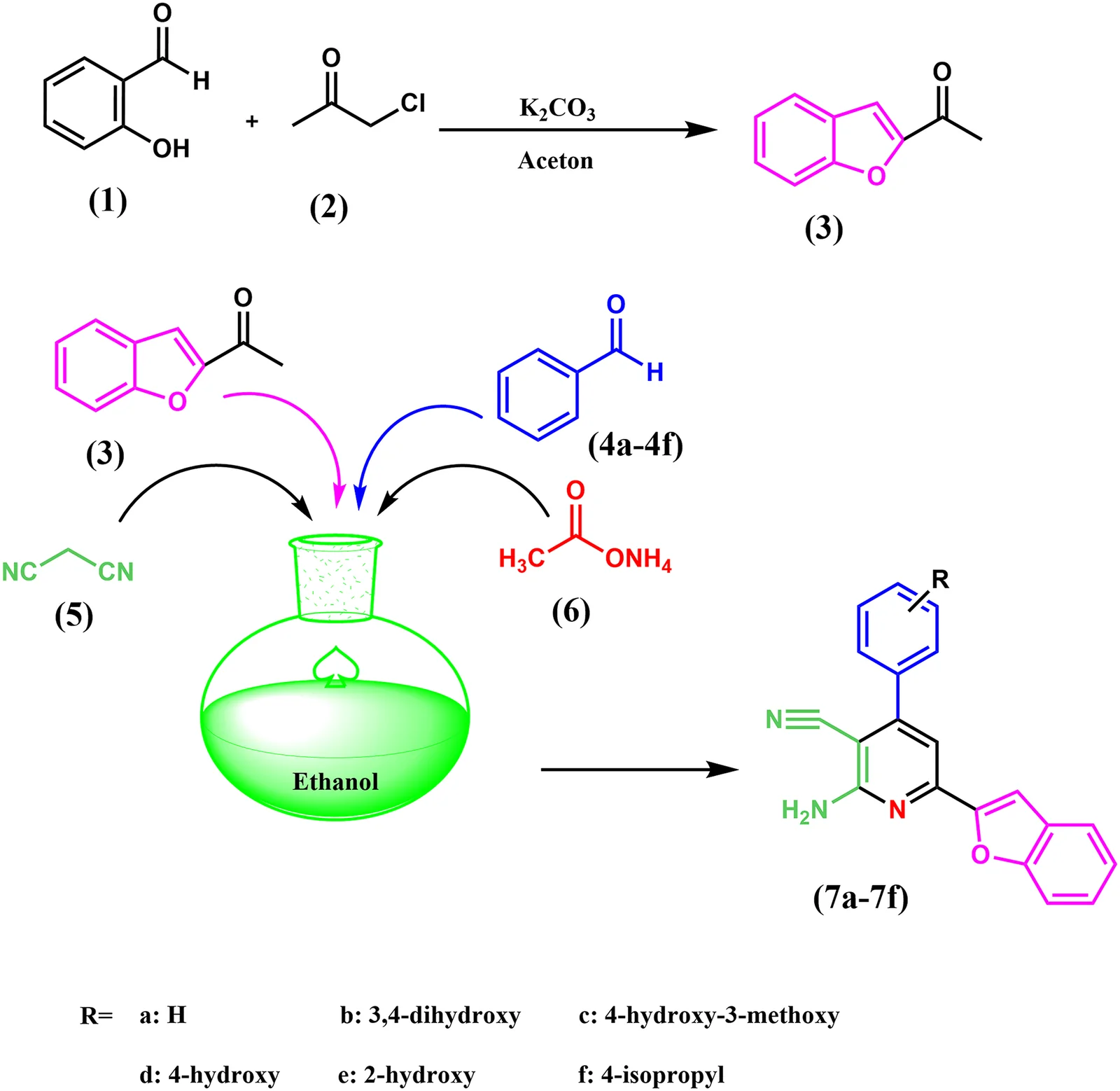
Synthesis route of pyridine-benzofuran hybrids

The pyridine-benzofuran hybrid compounds were prepared following the synthetic route depicted in Scheme 2. The synthesized compounds were characterized via melting point (mp) determination, as well as IR and NMR spectroscopy. The characterization data obtained for these compounds are detailed below:

#### 2-amino-6-(benzofuran-2-yl)-4-phenylnicotinonitrile (**7a**)

C_20_H_13_N_3_O; yield: 70%, white solid, mp: 120°C–122°C, IR (KBr, cm^−1^) ᴠ: 3,427 (NH_2_), 3,164 (Ar-CH), 2,335 (C≡N), 1,588 (C=N, pyridine), 1,483–1,447 (C=C aromatic), 1,299 (C–O), 1,211 (C–N); ^1^H NMR (400 MHz, DMSO-d_6_) δ: 8.83 (2H, NH_2_, br, s), 8.33 (1H, Ar-CH, pyridine-5), 7.92–7.91 (1H, Ar-CH, d, J = 8 Hz, benzofuran-7), 7.58–7.57 (2H, Ar-CH, m, benzofuran-5,6), 7.49–7.45 (2H, Ar-CH, m, benzene-2,6), 7.30–7.24 (3H, Ar-CH, m, benzene-3,4,5), 7.21–7.18 (1H, Ar-CH, d, J = 8.1 Hz, benzofuran-4), 6.97 (1H, Ar-CH, s, benzofuran-3); ^13^C NMR (100 MHz, DMSO-d_6_) δ: 161.2, 160.0, 158.3, 150.0, 135.2, 129.9, 128.9, 127.9, 126.4, 125.5, 124.3, 123.8, 120.0, 110.7, 108.5, and 101.9.

#### 2-amino-6-(benzofuran-2-yl)-4-(3,4-dihydroxyphenyl) nicotinonitrile (**7b**)

C_20_H_13_N_3_O_3_; yield: 80%, white solid, mp: 130.5°C–132°C, IR (KBr, cm^−1^) ᴠ: 3,492 (OH), 3,290 (NH_2_), 3,145 (Ar-CH), 2,283 (C≡N), 1,594 (C=N aromatic), 1,554–1,448 (C=C aromatic), 1,278 (C–O), 1,245 (C–O); ^1^H NMR (400 MHz, DMSO-d_6_) δ: 9.58 (1H, s, OH), 9.42 (1H, s, OH), 8.61 (2H, NH_2_, br, s), 8.15 (1H, Ar-CH, s, pyridine-5), 7.88–7.80 (2H, Ar-CH, m, dihydroxyphenyl-2,5), 7.46–7.41 (4H, Ar-CH, m, benzofuran-4,5,6,7), 7.02–7.00 (2H, Ar-CH, m, dihydroxyphenyl-6, furan-3); ^13^C NMR (100 MHz, DMSO- d_6_) δ: 160.4, 150.9, 135.5, 129.9, 129.8, 128.3, 128.1, 127.4, 127.1, 122.2, 120.0, 115.5, 110.0, 105.1, and 100.2.

#### 2-amino-6-(benzofuran-2-yl)-4-(4-hydroxy-3-methoxyphenyl) nicotinonitrile (**7c**)

C_21_H_15_N_3_O_3_; yield: 75%, white solid, mp: 140°C–141.1°C, IR (KBr, cm^−1^) ᴠ: 3,230 (OH, NH_2_, broad), 3,010 (Ar-CH), 2,280 (C≡N), 1,590 (C=N, pyridine), 1,548–1,412 (C=C aromatic), 1,225–11,124 (C–O), ^1^H NMR (400 MHz, DMSO-d_6_) δ: 8.86 (2H, NH_2_, br, s), 8.18 (1H, Ar-CH, s, pyridine-5), 7.85 (1H, Ar-CH, s, methoxyphenol-2), 7.81–7.80 (1H, Ar-CH, d, J = 8 Hz, methoxyphenol-5), 7.77–7.75 (1H, Ar-CH, d, J = 8 Hz, benzofuran-4), 7.60 (1H, Ar-CH, t, J = 8 Hz, benzofuran-5), 7.56–7.48 (2H, Ar-CH, m, benzofuran-6,7), 7.44–7.42 (1H, Ar-CH, m, methoxyphenol-6), 7.01–6.99 (1H, Ar-CH, s, benzofuran-3), 3.90 (3H, s, OCH3); ^13^C NMR (100 MHz, DMSO-d_6_) δ: 160.5, 160.0, 155.5, 155.4, 150, 146.5, 140.2, 135.1, 130.4, 125.3, 124.0, 122.1, 121.2, 120.0, 118.5, 117.4, 104.1, 100, and 56.5.

#### 2-amino-6-(benzofuran-2-yl)-4-(4-hydroxyphenyl) nicotinonitrile (**7d**)

C_20_H_13_N_3_O_2_; yield: 83%, white solid, mp: 100.2°C–102.0°C, IR (KBr, cm^−1^) ᴠ: 3,215 (OH, NH_2_, broad), 3,068 (Ar-CH), 2,261 (C≡N), 1,605-1,590 (C=C aromatic), 1,236-1,204 (C–O); ^1^H NMR (400 MHz, DMSO-d_6_) δ: 8.81 (2H, NH_2_, br, s), 8.22 (1H, Ar-CH, s, pyridine-5), 7.97–7.89 (1H, Ar-CH, d, J = 8 Hz, benzofuran-4), 7.87–7.85 (2H, Ar-CH, d, J = 8 Hz, phenyl-2,6), 7.82–7.80 (2H, Ar-CH, d, J = 8 Hz, phenyl-5,3), 7.52–7.48 (1H, Ar-CH, t, benzofuran-5), 7.48–7.42 (1H, Ar-CH, t, benzofuran-6), 7.06–7.03 (1H, Ar-CH, d, J = 8 Hz, benzofuran-7), 6.97 (1H, Ar-CH, s, benzofuran-3); ^13^C NMR (100 MHz, DMSO- d_6_) δ: 163.6, 160.0, 152.3, 152.2, 150.0, 134.5, 130.0, 130.9, 128.7, 127.6, 125.5, 122.8, 121.9, 120.0, 118.3, 107.6, and 106.5.

#### 2-amino-6-(benzofuran-2-yl)-4-(2-hydroxyphenyle) nicotinonitrile (**7e**)

C_20_H_13_N_3_O_2_; yield: 73%, white solid, mp: 139.7°C–141.2°C, IR (KBr, cm^−1^) ᴠ: 3,383 (OH, NH_2_, broad), 3,060 (Ar-CH), 2,341 (C≡N), 1,600 (C=N, pyridine), 1,560-1,436 (C=C aromatic), 1,275-1,264 (C–O), 1,227 (C–N, pyridine); ^1^H NMR (400 MHz, DMSO-d_6_) δ: 9.12 (1H, s, OH), 8.87 (2H, NH_2_, br, s), 8.31 (1H, Ar-CH, s, pyridine-5), 7.85–7.55 (7H, Ar-CH, m, benzofuran-4,5,6,7- phenyl-5,3,6), 6.84–6.80 (1H, Ar-CH, t, J = 8 Hz, phenyl-4), 6.65 (1H, Ar-CH, s, benzofuran-3); ^13^C NMR (100 MHz, DMSO- d_6_) δ: 165.4, 160.0, 155.5, 150.0, 148.3, 138.3, 133.7, 130.0, 129.1, 128.2, 127.3, 126.4, 125.5, 123.7, 121.9, 115.5, 112.8, and 100.0.

#### 2-amino-6-(benzofuran-2-yl)-4-(4-isopropylphenyl) nicotinonitrile (**7f**)

C_23_H_19_N_3_O_1_; yield: 83%, white solid, mp: 100.9°C–102.4°C, IR (KBr, cm^−1^) ᴠ: 3,248 (OH, NH_2_, broad), 3,060 (Ar-CH), 2,915 (aliphatic-CH), 2,299 (C≡N), 1,584 (C=N, pyridine), 1,551–1,429 (C=C aromatic), 1,286 (C–O), 1,247 (C–N, Pyridine); ^1^H NMR (400 MHz, DMSO- d_6_) δ: 8.55 (2H, NH_2_, br, s), 8.19 (1H, Ar-CH, s, pyridine-5), 7.87–7.84 (1H, Ar-CH, d, J = 8Hz, benzofuran-7), 7.81–7.79 (1H, Ar-CH, d, J = 8 Hz, benzofuran-4), 7.68–7.67 (2H, Ar-CH, d, J = 8Hz, isopropylbenzene-2,6), 7.59–7.45 (2H, Ar-H, m, isopropylbenzene-3,5), 7.44–7.42 (2H, Ar-H, m, benzofuran-5,6), 6.89 (1H, Ar-CH, s, benzofuran-3), 3.1–3.04 (1H, m, isopropyl-CH), 1.27 (6H, s, isopropyl-2CH3); ^13^C NMR (100 MHz, DMSO- d_6_) δ: 161.9, 160.0, 155.5, 151.9, 150.0, 137.3, 130.0, 135.5, 129.1, 128.2, 127.3, 126.4, 125.5, 124.6, 123.7, 122.8, 118.2, 117.3, 103.7, 100.0, 40, 32.8, and 22.8.

### Anti-inflammatory activity

**Figure 1:**
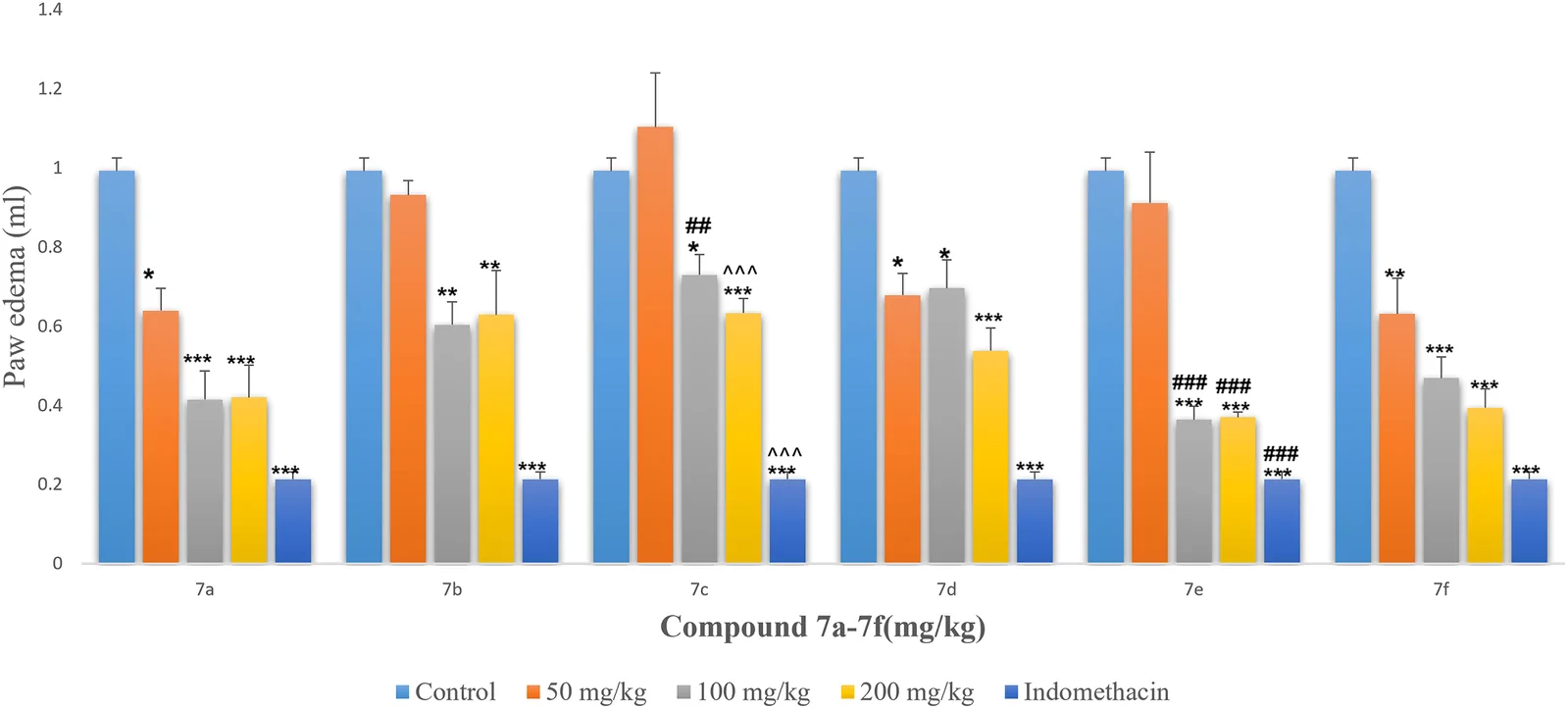
Anti-inflammatory activity of the synthesized compounds (**7a–7f**) in adult male rats Each compound was administered at doses of 50, 100, and 200 mg/kg, 30 min before sub-plantar injection of carrageenan into the right hind paw. Indomethacin (10 mg/kg, intraperitoneally) was used as the reference drug. Paw volume (in mL) was measured immediately before carrageenan injection and again 4 h post-injection using a water plethysmometer. Data are expressed as mean ± standard error of the mean (SEM) for each group (*n* = 6). Statistical significance was assessed using one-way ANOVA followed by Scheffe post hoc test. ∗*p* < 0.05, ∗∗*p* < 0.01, ∗∗∗*p* < 0.001 compared with the control group; ^##^*p* < 0.01 and ^###^*p* < 0.001 compared with the group that received 50 mg/kg and ^ˆˆˆ^*p* < 0.001 in comparison with the group that received 100 mg/kg.

The synthesized pyridine-benzofuran hybrid compounds (**7a**–**7f**) were evaluated for their anti-inflammatory activity using the carrageenan-induced paw edema model.^24^^,^^25^ The groups included a control group receiving vehicle only; three experimental groups receiving intraperitoneal injections of any of the synthesized compound (**7a**–**7f**) at doses of 50, 100, and 200 mg/kg; and a standard group treated with indomethacin at a dose of 10 mg/kg. The obtained results are in Figure 1.

### Molecular docking study

Molecular docking study of the synthesized compounds (**7a**–**7f**) was conducted using AutoDock 4.2 software.^26^ For this purpose, the crystallographic structure of COX-2 (PDB: 6BL3 and resolution: 2.2 Å) was used as the target and the binding interactions and affinities of the synthesized ligand were evaluated *in silico*. The results of the analysis are summarized in Table 1.

**Table 1:** Free binding energy (kcal/mol), interactions, as well as inhibition constants (Ki) of synthesized compounds with COX-2 enzyme calculated by AutoDock

Compound	Binding energy^a^	Inhibition constants	H bond	H bond distance	Hydrophobic interaction
**7a**	-8.14	1.08 μM^b^	ND	ND	Leu531, Ser530, Val349, Ser353, Trp387, Tyr348, Gln192, His90, Tyr355, Ile517, Ala516, and Arg513
**7b**	-8.03	1.29 μM^b^	Tyr385, Ser530, Tyr355, and Leu352,	2.09 Å, 2.20 Å, 1.75 Å 3.04 Å	Tyr348, Phe518, Arg120, Val116, Ser119, His90, and Val523
**7c**	-7.49	3.24 μM^b^	ND	ND	Glu524, His90, Val89, Arg120, Tyr355, Ala527, Val349, and Ala527
**7d**	-7.4	3.74 μM^b^	Tyr385, Tyr355, and Leu352,	2.09 Å 1.70 Å 3.10 Å	Val523, Ala527, Ser530, Val116, Arg120, Ser119, His90, Val349, Ser353, Phe518, and Tyr348
**7e**	-7.92	1.55 μM^b^	Tyr385, Leu352, and Val523,	1.75 Å 3.07 Å 2.21 Å	Val116, Arg120, His90, Ser530, Glu524, and Ala527
**7f**	-8.59	502.8 nM^c^	ND	ND	Val116, Arg120, Ala527, Val349, Leu352, Val523, Tyr355, His90, Arg513, Ala516, and Phe518
Indomethacin	-8.52	564.31 nM^c^	ND	ND	Arg513, Tyr355, Ser353, Val523, Ala527, Val349,His90, Met522, and Gln192
Celecoxib	-10.92	9.82 nM^c^	Phe518, Arg513, and His90	2.00 Å 2.30 Å 2.17 Å	Leu352, Ser353, Tyr355, Leu359, Val349, and Gln192

a kcal/mol.

b Nanomolar.

c Micromolar.

### Molecular dynamics simulations

**Figure 2:**
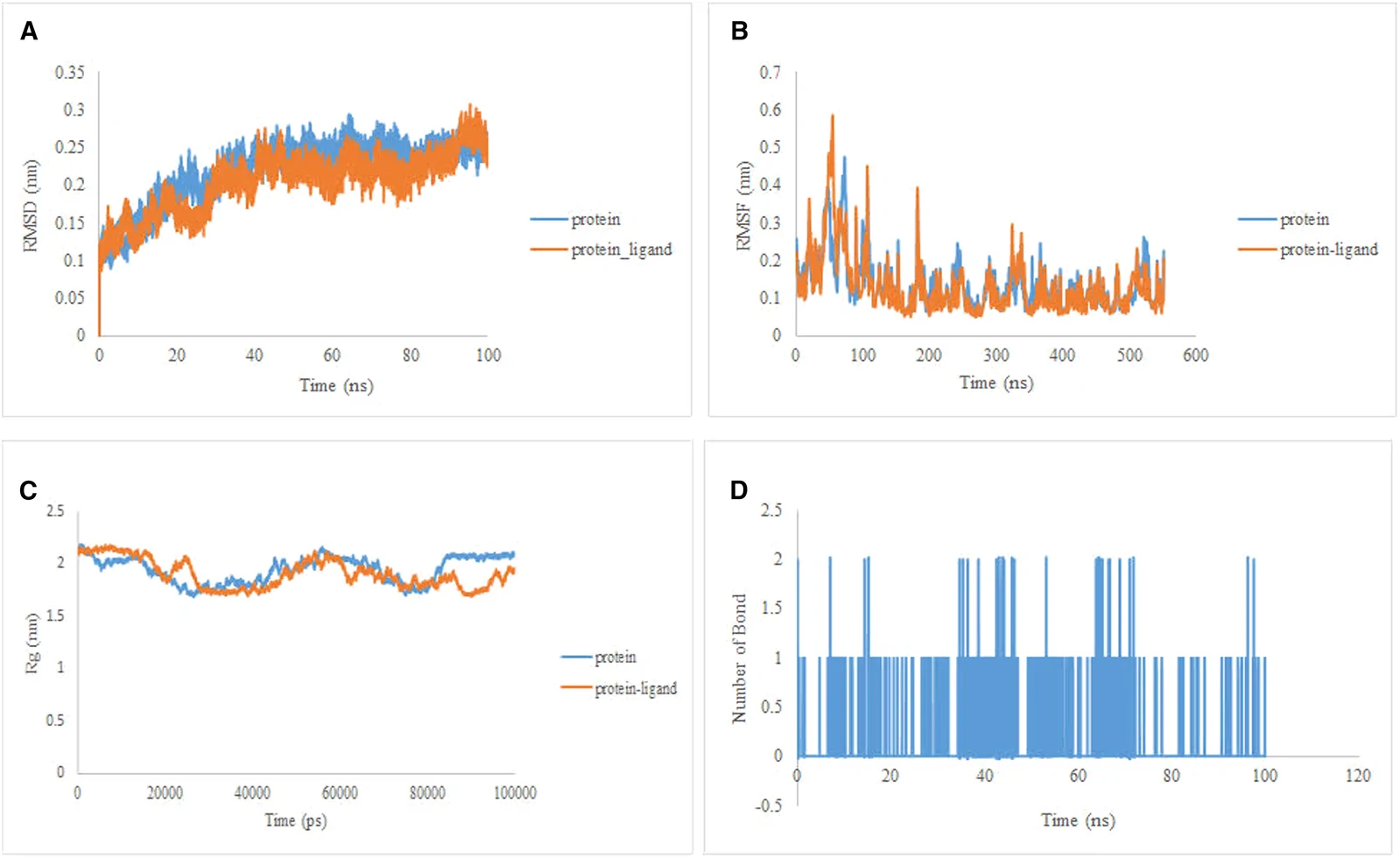
MD simulation of 7a-COX-2 complex in 100 ns Molecular dynamics (MD) simulation results of the **7a–COX-2** complex over a 100 ns trajectory. The panels illustrate the structural stability and conformational dynamics of the complex: (A) Root mean square deviation (RMSD); (B) Root mean square fluctuation (RMSF) per residue; (C) Radius of gyration (Rg); and (D) Hydrogen bond interactions between the ligand and the receptor.

Based on the correlation between the *in vivo* and *in silico* results, compounds **7a** and **7f** emerged as the top-performing candidates of this study. However, given that the biological efficacy and docking profiles of **7a** and **7f** were remarkably comparable, we selected compound **7a** as the representative lead to undergo further MD simulations. For this purpose, changes and stability of the structure of the COX-2 enzyme in the **7a**-bound state were investigated by root-mean-square deviation (RMSD), root mean square fluctuation (RMSF), *R*g analysis, and modeling hydrogen bonds (Figure 2).

## Discussion

In this study, based on the anti-inflammatory activity of benzofuran derivatives and naturally occurring aldehydes, new pyridine-benzofuran hybrid derivatives were synthesized and evaluated for their anti-inflammatory effects. The initial design of these hybrids was not strictly target-driven; instead, the compounds were designed based on a pharmacophore hybridization strategy: merging the known anti-inflammatory properties of benzofuran scaffolds and natural aldehydes to potentially preserve or enhance this activity, while ensuring synthetic feasibility.

### Preparation of novel pyridine-benzofuran derivatives (**7a–7f**)

The synthesis of 1-(benzofuran-2-yl) ethan-1-one (**3**) is achieved via a nucleophilic condensation reaction. Initially, the hydroxyl group of salicylaldehyde (**1**) is deprotonated in the presence of potassium carbonate (K_2_CO_3_), which acts as a base. The resulting phenolate anion then attacks the electrophilic α-carbon of chloroacetone (**2**), leading to the displacement of the chlorine atom and formation of a new C–O bond. Subsequently, an intramolecular condensation occurs between the ortho-positioned aldehyde group and the ester moiety, resulting in the formation of a five-membered ring and yielding compound (**3**).^27^

In the next stage, deprotonation of malononitrile (**5**), which contains two electron-withdrawing cyano groups, leads to the formation of an active carbon nucleophile. This nucleophile attacks the carbonyl carbon of the aldehyde derivatives (**4a–4f**), forming a β-hydroxy nitrile intermediate via an aldol-type addition. The intermediate then undergoes dehydration, eliminating a hydroxyl group and creating a C=C double bond between the α- and β-carbons, resulting in an α, β-unsaturated compound. The deprotonated acetyl benzofurane reacted with the α, β-unsaturated intermediate and subsequently, ammonium acetate, which serves as an amine source leads to the formation of an imine bond. Finally, intramolecular cyclization resulted in the formation of the pyridine ring and the novel hybrid benzofuran-pyridine derivatives (**7a**–**7f**) were obtained.

### Characterizations of synthesized compounds

To evaluate the structural integrity of the pyridine-benzofuran hybrids and identify their functional groups, IR spectroscopy was employed. The main vibrational bands of compounds **7a**–**7f** exhibited absorptions in the range of 3,215–3,492 cm^−1^, corresponding to the presence of NH and OH groups, mostly appearing as broad peaks. The absorptions observed in the region 3,010–3,164 cm^−1^ were attributed to aromatic C-H stretching, while peaks in the range 2,261–2,341 cm^−1^ indicated the presence of nitrile (C≡N) groups. Vibrations in the 1,588–1,600 cm^−1^ region confirmed the presence of C=N within the pyridine ring. Absorptions between 1,412 and 1,605 cm^−1^ were associated with the aromatic framework, and bands in the 1,202–1,299 cm^−1^ region corresponded to C-O stretching of the furan ring. The IR spectra of compounds **7a–7f** are presented in Figures S1–S6 of the Supporting Information.

To investigate the proton environments and confirm the structures of the synthesized compounds (**7a-7f**), ^1^H NMR spectroscopy was conducted. The observed signals were consistent with the proposed structures and confirmed the presence of various functional groups. Signals in the range of 8.61–8.86 ppm corresponded to NH_2_ protons, mainly appearing as broad peaks. Aromatic protons of the pyridine ring were observed between 8.13 and 8.33 ppm, while other aromatic protons were typically observed in the 6.65–6.99 ppm region. A signal around 3.8 ppm confirmed the presence of a methoxy group in **7c** and isopropyl protons in compound **7f** were detected at 3.04 (CH) and 1.5 (2CH_3_) ppm.

**Figure 3:**
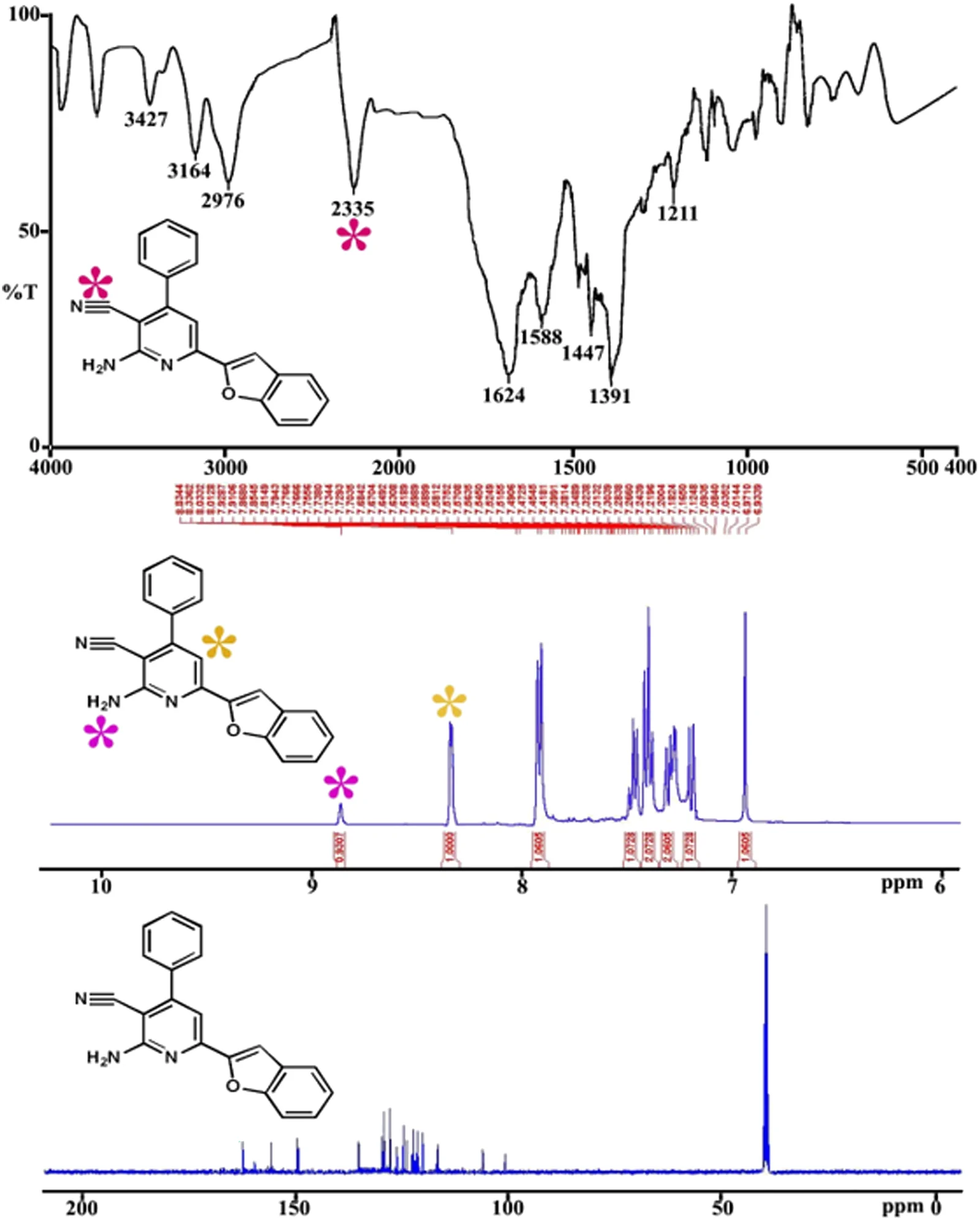
IR and NMR spectra of compound **7a**

To identify the carbon atoms within the molecular framework, ^13^C NMR spectroscopy was performed. A signal at 56.5 ppm was attributed to the methoxy group in compound **7c**. Signals between 22.8 and 32.8 ppm confirmed the presence of isopropyl carbons in compound **7f**. Other signals corresponding to the aromatic carbons and the nitrile group in the ^13^C NMR spectrum were consistent with the proposed structure. As an example, IR and NMR spectra of compound **7a** are shown in Figure 3. The ^1^H NMR and ^13^C NMR spectra of compounds **7a–7f** are presented in Figures S7–S18 of the supplemental information.

### Anti-inflammatory activity

To assess the anti-inflammatory effects of the synthesized compounds (**7a**–**7f**), the carrageenan-induced paw edema model was employed in adult male rats. This model exhibits a biphasic inflammatory response: the early phase (0–1.5 h) is primarily mediated by histamine and serotonin, while the late phase (1.5–6 h) is mainly driven by the production of prostaglandins and nitric oxide, along with the activation of the COX-2 pathway.^25^

Thirty minutes after administration of treatments—saline for the negative control group, indomethacin for the positive control group, and compounds **7a**–**7f** for the treatment groups—carrageenan was injected into the sub-plantar region of the right hind paw. Paw volume was measured using a water plethysmometer immediately prior to carrageenan injection and 4 h post-injection. The difference between these measurements was calculated as paw edema and compared across the groups (Figure 1).

Significant paw edema was observed in the negative control group. In contrast, treatment with compounds **7a**–**7f** exhibited a statistically significant reduction in paw edema volume, indicating the anti-inflammatory potential of these compounds. Data are expressed as mean ± standard deviation, and statistical significance was determined using ANOVA (*p* value). The synthesized compounds (**7a**–**7f**) demonstrated varying degrees of anti-inflammatory activity. Among the tested compounds, compounds **7a** and **7e** with 61.2% and 63.2% inhibition at a dose of 100 mg/kg (∗∗∗*p* < 0.001), and compounds **7a**, **7e**, and **7f**, with 57.6%, 62.7%, and 60.3% inhibition at a dose of 200 mg/kg (∗∗∗*p* < 0.001), were the best-performing compounds studied compared with indomethacin (78.5% inhibition) of the reference drug. Compound **7e** showed negligible efficacy at the dose of 50 mg/kg; in contrast, compounds **7a** and **7f**, administered at the same dose, achieved 35.5% and 36.5% inhibition, respectively. Conversely, compound **7c** at a dose of 50 mg/kg did not exhibit any significant anti-inflammatory activity and even produced a pro-inflammatory effect (-11.1% inhibition). At 100 mg/kg, the compound displayed only moderate efficacy (∗∗*p* < 0.01), whereas at 200 mg/kg, it exerted a markedly higher anti-inflammatory effect (∗∗∗*p* < 0.001) compared with its lower doses. Compounds **7d** and **7b** exhibited moderate efficacy (45.7%–36.4%, ∗∗∗*p* < 0.001 and ∗∗*p* < 0.01 inhibition) at higher doses, but lacked significant activity at lower doses. Regarding dose dependency, compounds **7a**, **7e**, and **7f** displayed consistent pharmacodynamics responses. Notably, compound **7f** showed statistically significant effects (∗∗*p* < 0.01) across all tested doses. Compound **7e** reached its maximal efficacy at 100 mg/kg (^###^*p* < 0.001), and increasing the dose to 200 mg/kg did not yield a significant improvement, suggesting a plateau in the dose-response relationship. In conclusion, compounds **7a** and **7f** are proposed as lead candidates for anti-inflammatory drug development due to their high efficacy and strong statistical significance. In contrast, the other synthesized compounds require structural optimization to enhance their pharmacological profiles.

It is important to note that while the synthesized hybrids demonstrated significant anti-inflammatory activity, their efficacy remained lower than that of the reference drug, indomethacin. The rationale behind pharmacophore hybridization is based on the hypothesis that merging bioactive moieties may enhance biological activity; however, this requires empirical validation, as experimental outcomes can vary. This study provides a valuable preliminary screening, demonstrating that these specific benzofuran-natural aldehyde architectures possess inherent anti-inflammatory potential. These findings establish the synthesized hybrids as promising lead compounds for future structural optimization aim at improving potency.

### Molecular docking study

The carrageenan-induced paw edema model is a robust, gold standard *in vivo* assay that evaluates the net, holistic anti-inflammatory efficacy (which inherently encompasses downstream effects like COX-2 modulation) within a complex physiological system. Subsequently, upon confirming the *in vivo* anti-inflammatory activity, molecular docking studies were conducted to explore COX-2 as a probable mechanistic target. In this study, the binding interactions and their affinities were evaluated *in silico*. The results of the analysis are summarized in Table 1.

**Figure 4:**
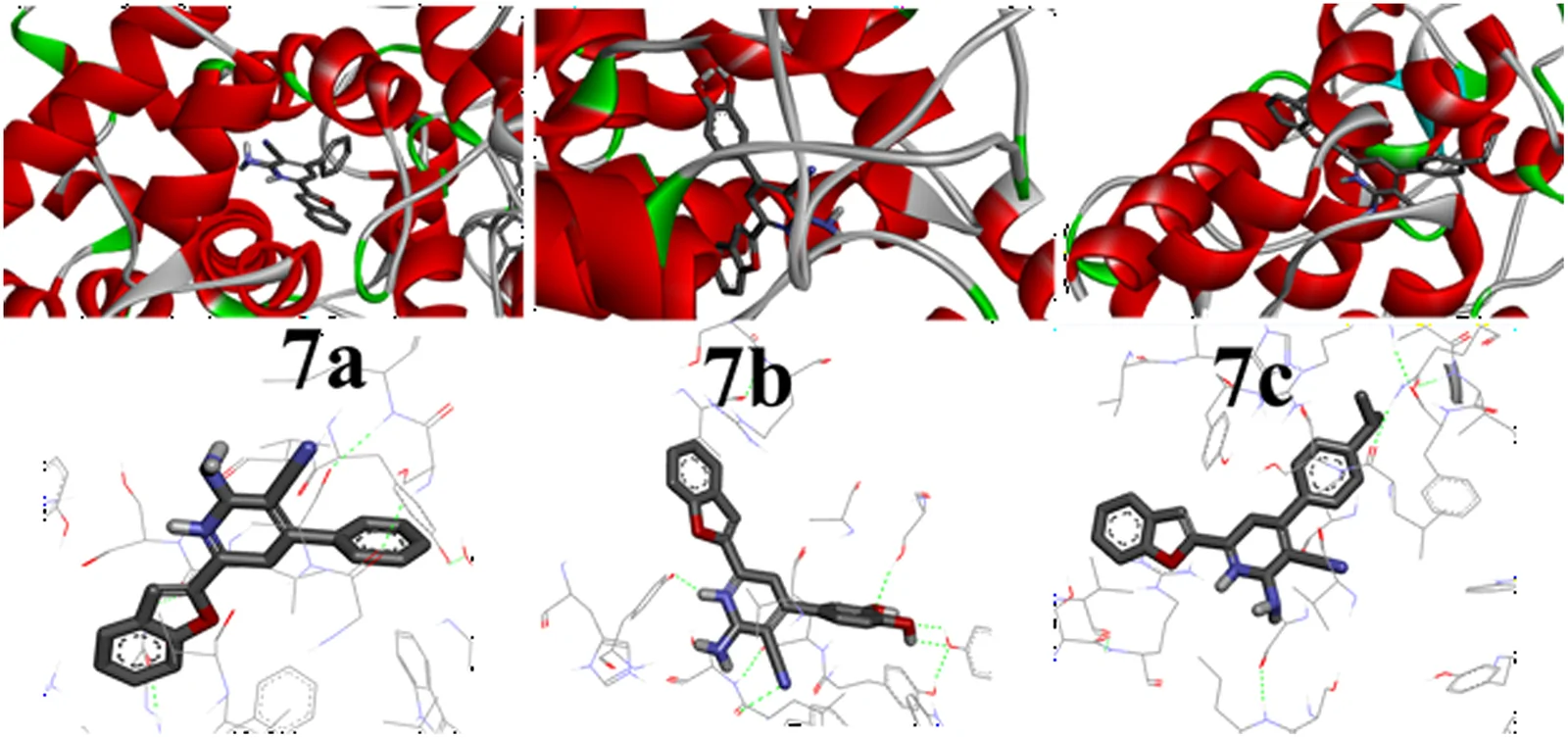
Docking binding modes of 7a, 7b and 7f in COX-2 Predicted binding modes of compounds **7a**, **7b**, and **7f** within the active site of the COX-2 enzyme. The binding poses were generated using AutoDock4, illustrating the spatial orientation and key interactions of **(a)** compound **7a**, **(b)** compound **7b**, and **(c)** compound **7f** with the active site residues.

Docking simulations revealed that compounds **7a**–**7f** exhibited favorable binding affinities toward the active site of COX-2 compared to the co-crystal ligand. However, notable differences were observed in their binding energies and interaction profiles. Among the tested compounds, compound **7f** showed the highest binding affinity to COX-2, with a ΔG value of -8.59 kcal/mol and an inhibition constant (Ki) of 502.8 nM. Although it lacked hydrogen bonding interactions, it formed extensive hydrophobic contacts with key amino acid residues such as His90, Tyr355, Arg120, and Phe518, indicating a stable and strong binding tendency. Compounds **7a** and **7b** also demonstrated favorable binding energies of -8.03 and -8.14 kcal/mol, respectively, and were considered among the highest-affinity ligands. Notably, compound **7b** formed effective hydrogen bonds with Tyr385, Ser530, and Leu352, contributing to the stability of the complex. The presence of hydroxyl in compound **7e** facilitated the formation of hydrogen bonds with Tyr385, Leu352, and Val523 residues, stabilizing its interaction with COX-2. Additionally, its broad hydrophobic interactions with the active site suggest a moderate to favorable inhibitory potential. In contrast, compounds **7c** and **7d** exhibited relatively weaker binding profiles, with Δ*G* values of -7.49 and -7.40 kcal/mol, and Ki values exceeding 3 μM. Compound **7c** lacked any hydrogen bonding interactions and relied solely on weaker hydrophobic contacts. Although compound **7d** formed hydrogen bonds, its high Ki and lower binding energy indicated reduced efficacy. Figure 4 depicts the optimal binding orientations of the **7a**, **7e**, and **7f** in the active site of COX-2. These results suggest that compounds **7a** and **7f**, due to their high affinity for COX-2 and consistency with the biological data, possess strong potential for development as selective anti-inflammatory agents.

### Molecular dynamics simulations

Based on the correlation between the *in vivo* and *in silico* results, compounds **7a** and **7f** emerged as the top-performing candidates of this study. However, given that the biological efficacy and docking profiles of **7a** and **7f** were remarkably comparable, we selected compound **7a** as the representative lead to undergo further MD simulations. For this purpose, changes in the structure of the COX-2 enzyme in the **7a**-bound state were analyzed by RMSD analysis. The results revealed that the system reached conformational stability after approximately 30 ns, with fluctuations remaining within an acceptable range (≤3 Å), and the average RMSD of the complex throughout the simulation was nearly identical to that of the unbound protein (Figure 2A), indicating structural stability of the enzyme during interaction with **7a**.^28^

Residue-level flexibility was assessed using RMSF analysis. The average RMSF change in the apo protein is approximately 0.17 nm, whereas for the ligand-bound protein it is about 0.13 nm. This slight reduction in RMSF may indicate enhanced structural stability and restricted mobility of the enzyme’s amino acids in the ligand-bound state compared with the ligand-free state (Figure 2B).^29^

In MD studies, one of the most important criteria for evaluating protein stability and flexibility is the assessment of protein compactness through *R*g analysis. In this study, the average *R*g value for the apo protein is approximately 10 Å, while for the protein in the presence of the ligand, it is 9 Å. Although the *R*g of the complex was marginally higher during 14–27 ns, it remained consistently lower before and after this period. However, before and after this period the *R*g decreases throughout the simulation. This reduction, and the consequent increase in protein compactness, indicates effective protein-ligand interaction and greater structural stability compared to the apo state (Figure 2C).^30^

Another crucial factor for investigating intermolecular interactions and structural stability is the hydrogen bonds formed between the ligand and the protein throughout the simulation time. Hydrogen bonds not only play a significant role in protein-ligand interactions but are also essential for the folding and unfolding processes of proteins, which rely on the hydrogen bonds within the protein structure.^31^ MD simulation studies, by analyzing donor-acceptor pair distributions, are capable of modeling hydrogen bonds. The results of this study indicate the formation of at least one hydrogen bond during the simulation time between the ligand and the protein which led to the proper orientation of the compound against the protein (Figure 2D). According to the obtained results, compound **7a** may remain stable within the active site cavity of the COX-2 enzyme over a 100-ns timescale and thereby inhibit this enzyme, providing a structural basis for its inhibitory activity.

In conclusion, a series of novel pyridine-benzofuran hybrid compounds were synthesized through a simple, mild, and efficient MCR and structurally characterized using IR, ^1^H NMR, and ^13^C NMR spectroscopy. The *in vivo* anti-inflammatory activity was evaluated in the carrageenan-induced paw edema model in adult male rats. Among the tested derivatives, compounds **7a** and **7e** emerged as the most potent agents, exhibiting dose-dependent inhibition of 35.5% and 36.6% at 50 mg/kg, 61.2% and 53.6% at 100 mg/kg, and 57.6% and 60.3% at 200 mg/kg, respectively (∗∗∗*p*∗ < 0.001). Subsequently, upon confirming the *in vivo* anti-inflammatory activity, molecular docking studies were conducted to explore COX-2 as a probable mechanistic target and compounds **7a**, **7b**, and **7f** exhibited the most favorable binding energies (below -8 kcal/mol). Based on the combined *in vivo* and *in silico* results, compound **7a** was selected for MD simulation. The results indicated that the ligand-protein complex remained structurally stable throughout the 100-ns simulation trajectory, confirming the formation of a stable and effective protein-ligand interaction.

### Limitations of the study

Despite the promising preliminary *in vivo* anti-inflammatory activity of the synthesized hybrids, several limitations of the current study must be acknowledged to provide a balanced interpretation of the results. First, the effective doses required to achieve significant edema inhibition were relatively high compared with the reference drugs, indicating a clear need for further structural optimization to enhance potency. Second, this study relies on *in vivo* phenotypic evaluation and computational modeling; therefore, direct *in vitro* COX-2 inhibition assays and isoform selectivity studies (COX-1 vs. COX-2) were not conducted in this initial phase and remain essential for future mechanistic validation. Future investigations will focus on the structural modification of these lead compounds to improve their efficacy at lower doses, followed by comprehensive *in vitro* enzymatic, selectivity, and pharmacokinetic profiling.

## Resource availability

### Lead contact

Requests for further information and resources should be directed to and will be fulfilled by the lead contact, Parvin Asadi (asadi@pharm.mui.ac.ir).

### Materials availability

This study did not generate new unique reagents.

### Data and code availability

• All data reported in the paper are available from the lead contact upon request.
• This paper does not report original code.
• Any additional information required to reanalyze the data reported in this paper is available from the lead contact upon request.

## Acknowledgments

We gratefully acknowledge the financial support of 10.13039/501100003970Isfahan University of Medical Sciences, Isfahan, Iran with project no. 3403912.

## Author contributions

Conceptualization, P.A., V.H., and G.K.; methodology, P.A., G.K., and V.H.; investigation, H.S., P.A., V.H., and G.K.; writing – original draft, H.S.; writing – review and editing, P.A. and V.H.; funding acquisition, P.A.; supervision, P.A. and V.H.

## Declaration of interests

The authors declare no competing interests.

## Declaration of generative AI and AI-assisted technologies in the writing process

The Qwen artificial intelligence model was used exclusively for linguistic refinement and English language editing of this manuscript. All scientific content, data analysis, and interpretation remain the sole responsibility of the authors.

## STAR★Methods

### Key resources table

**Table:** 

REAGENT or RESOURCE	SOURCE	IDENTIFIER
**Chemicals, peptides, and recombinant proteins**		

malononitrile	Merck	806189
salicylaldehyde	Merck	S356-100G
chloroacetone	Merck	802603
vanillin	Merck	108510
benzaldehyde	Merck	801756
4-Hydroxybenzaldehyde	Merck	804536
Cuminaldehyde	Merck	135178
ammonium acetate	Merck	101116

**Experimental models: Organisms/strains**		

Rattus norvegicus (Wistar rat), Male	Animal facility, School of Pharmacy, Isfahan University of Medical Sciences	In-house bred

**Software and algorithms**		

AutoDock 4	The Scripps Research Institute	Version4.2.3.; https://autodock.scripps.edu/
HyperChem	HyperCube, Inc.	Version 8 http://www.hypercubeusa.com/
ChemDraw	PerkinElmer Informatics	Version 12; https://revvity.com/
GROMACS	GROMACS development team	Version 2022; https://www.gromacs.org

### Experimental model and study participant details

Male Wistar rats (weight: 180–200 g; age: 8–10 weeks) were obtained from the Animal House Facility of Isfahan University of Medical Sciences, Isfahan, Iran. Animals were housed in standard polycarbonate cages under controlled environmental conditions (temperature: 22 ± 2°C; humidity: 50 ± 10%; 12-h light/dark cycle) with free access to standard rodent chow and water *ad libitum*. Animals were allowed to acclimate to the laboratory environment for at least 7 days prior to the initiation of experiments. All procedures were performed in accordance with the National Institutes of Health Guide for the Care and Use of Laboratory Animals (NIH Publications No. 80-23, revised 1996) and were approved by the Animal Ethics Committee of Isfahan University of Medical Sciences (Approval Code: IR.MUI.AEC.1404.009). All efforts were made to minimize animal suffering and to reduce the number of animals used. Animals were euthanized immediately upon completion of each experiment by CO_2_ inhalation.

In this study, only male rats were used to avoid hormonal fluctuations associated with the estrous cycle that could confound the inflammatory response. The influence of sex on the observed outcomes was not evaluated, which represents a limitation of the present study and should be addressed in future investigations to improve the generalizability of the findings.

### Method details

#### Materials and methods

Commercial compounds for the synthesis of the desired derivatives were obtained from Merck and Sigma. Spectroscopic methods were employed for the structural elucidation and characterization of the synthesized compounds. ^1^HNMR and ^13^CNMR spectra were recorded using an NMR spectrometer (Bruker, Germany) with tetramethyl silane (TMS) as the internal standard, and coupling constants (J values) were estimated in Hertz (Hz). Infrared (IR) spectra were obtained as KBr pellets using a Perkin-Elmer 680 IR spectrometer (Japan). Melting points were determined using an Electrothermal 9200 melting point apparatus (UK) and are uncorrected. Thin-layer chromatography (TLC) was performed to monitor reaction progress and assess the purity of the synthesized compounds, using Merck silica gel 60 F_254_ plates (Germany). All chemical structures were drawn, and the corresponding molecular formulas were calculated and verified using ChemDraw software.

#### Preparation of 1-(benzofuran-2-yl) ethan-1-one (**3**)

Initially, anhydrous potassium carbonate (24 mmol, 3.35 g) was added to a solution of salicylaldehyde (**1**, 1.1 g, 8.1 mmol) in acetone (15 mL). Upon deprotonation of the acidic hydrogen from salicylaldehyde, a pale-yellow color appeared in the reaction medium, indicating activation of the compound. At this stage, chloroacetone (**2**, 0.81 g, 8.1 mmol) was added to the mixture, and the reaction was refluxed for 12 hours. After confirming the completion of the reaction by TLC, the mixture was cooled to room temperature and filtered to remove potassium carbonate. The resulting clear solution was concentrated using a rotary evaporator, and then recrystallized in diethyl ether and *n*-hexane. The desired product (**3**) was obtained as crystalline solid (yield: 90%).^27^

#### Preparation of 2-amino-6-(benzofuran-2-yl)-4-phenylnicotinonitrile derivatives (**7a–7f**)

A mixture of aromatic aldehyde (**4**, 5mmol), malononitrile (**5**, 0.3 g, 5mmol), **3** (0.7 g, 5mmol) and ammonium acetate (**6**, 0.3 g, 5 mmol) was prepared in absolute ethanol (15 mL). The reaction mixture was refluxed for 13 h and the reaction was monitored by TLC on silica gel plates. Methanol and chloroform (1:10) were used as the mobile phase and spots were visualized by UV irradiation. After the completion of the reaction, the mixture was cooled to room temperature (RT), in all cases (except **7a**), the solid product was filtered, washed with ethanol, and air-dried, whereas benzaldehyde derivative (**7a**) was extracted with chloroform.

#### Evaluation of the anti-inflammatory effects

The synthesized pyridine-benzofuran hybrid compounds (**7a-7f**) were evaluated for their anti-inflammatory activity using the carrageenan-induced paw edema model.^24^^,^^25^ To assess the effects of each of the synthesized compounds (**7a-7f**), a total of 20 groups (six rats in each group) were used. The groups included: a control group receiving vehicle only, three experimental groups receiving intraperitoneal injections of any of the synthesized compound (**7a-7f**) at doses of 50, 100, and 200 mg/kg, and a standard group treated with indomethacin at a dose of 10 mg/kg.

Thirty minutes after drug administration, 100 μL of 1% carrageenan solution (w/v) was injected into the plantar surface of the right hind paw of each rat. Paw volume was measured using a water plethysmometer immediately before carrageenan injection and again four hours post-injection. The difference in paw volume was recorded as an indicator of edema and used to compare the anti-inflammatory efficacy across the groups. For the preparation of the synthesized compounds (**7a-7f**) before injection, a uniform suspension was formulated using Tween 80 in saline as the suspending agent, and the resulting suspension was administered intraperitoneally.

#### Molecular docking study

Molecular docking study of the synthesized compounds (**7a-7f**) was conducted using AutoDock 4.2 software.^26^ For this purpose, the crystallographic structure of COX-2 (PDB ID: 6BL3, resolution: 2.2 Å) was retrieved from the Protein Data Bank (PDB). In this study, indomethacin was selected as the reference ligand for docking validation solely because it is the native co-crystallized ligand in the utilized crystal structure, ensuring accurate computational validation. This choice was based on structural and methodological grounds, not on the therapeutic profile of indomethacin.

Before docking, protein preparation was performed. Co-crystallized ligands and crystallographic water molecules were removed from the protein structures. Polar hydrogens and Kollman charges were added using AutoDock Tools. The processed protein structures were then converted to PDBQT format for docking. For ligand preparation, the synthesized compounds (**7a-7f**) were initially drawn using ChemDraw 8.0 and geometrically optimized using HyperChem software. The most stable conformations were selected and converted to PDBQT format after adding polar hydrogen and Gasteiger charges. In the next step, the Grid Box was defined at x = −47.678, y = −16.923, z = 35.116 and docking simulations were carried out using AutoGrid and AutoDock software, targeting the active sites of the enzymes. Following the docking procedure, the outputs including ligand conformations, clustering results, binding energies, and types of interactions between ligands and proteins, were extracted and analyzed.

The molecular docking and molecular dynamics (MD) simulations were conducted using the crystal structure of COX-2 co-crystallized with indomethacin (PDB ID: 6BL3). The indomethacin-bound conformation was specifically selected for evaluating the designed hybrids for two reasons. First, indomethacin served as the positive control in the *in vivo* pharmacological assays, allowing for direct *in silico*–*in vivo* correlation. Second, from a structural perspective, the hybrids compounds exhibit pharmacophores similar to indomethacin, which anchors deeply into the main hydrophobic channel. Using the indomethacin-bound structure (which features the native, closed side-pocket conformation) provides a more rigorous and realistic template for assessing the deep-penetrating binding mode of these hybrids, avoiding potential artifacts associated with the artificially 'open' side pocket observed in celecoxib-bound structures.

#### Molecular dynamics study

GROMACS 2022 software using the GROMOS force field^32^ was used to run a 100 ns MD simulation. The initial framework for studying the MD complex, the **7a**-COX2 complex, was selected based on docking studies. This choice was motivated by the fact that **7a** was among the top three compounds in both the biological studies and the docking results. Topology and parameter files for the ligand were prepared via the SwissParam server.^33^ The systems were solvated in cubic boxes filled with SPC-modeled water molecules.^34^ For energy minimization, the steepest descent method was utilized initially to release steric clashes.^35^ Simulation conditions were maintained at 1 atm pressure and 300 K using a Parrinello–Rahman barostat and a Nosé–Hoover thermostat, respectively.^36^ The systems were energy-minimized using the steepest descent algorithm to remove steric clashes, followed by 10 ns equilibration phases under constant volume and temperature (NVT) and subsequently 10 ns under constant pressure and temperature (NPT). For reproducibility, energy-time series were examined to assess convergence and stability. The MD simulations were conducted for 100 ns with a time step of 2 femtoseconds. Short-range non-bonded interactions cutoffs were set at 1 nm, and long-range electrostatics were calculated using the particle mesh Ewald (PME) method.^37^ The LINCS algorithm^38^ was applied to constrain bond lengths.

### Quantification and statistical analysis

For the analysis of data obtained from the carrageenan-induced paw edema assay, the paw volume of each animal was measured at time zero (prior to carrageenan injection) and at predetermined time intervals following injection. The change in paw volume was considered as the quantitative index of edema severity and inflammatory response. Data for each experimental group (*n* = 6) were expressed as mean ± standard error of the mean (SEM). To compare the different experimental groups (control, reference drug indomethacin, and various doses of the synthesized compounds), one-way analysis of variance (ANOVA) was employed. Statistical significance was defined at *p*-values of < 0.05, < 0.01, and < 0.001.
