# Supplementary material for: Green synthesis of pyridine-benzofuran hybrids with anti-inflammatory activity alongside in silico study
Source: iScience. 2026 Sep 7;29(9):117389. doi: 10.1016/j.isci.2026.117389 (PMC13577876; doi:10.1016/j.isci.2026.117389)
Supplement: Supplementary file 1 [file mmc1.pdf]

iScience, Volume *29*

Supplemental information

Green synthesis of pyridine-benzofuran hybrids with anti-inflammatory activity alongside *in silico* study

Haniye Salehi, Valiollah Hajhashemi, Ghadamali Khodarahmi, and Parvin Asadi

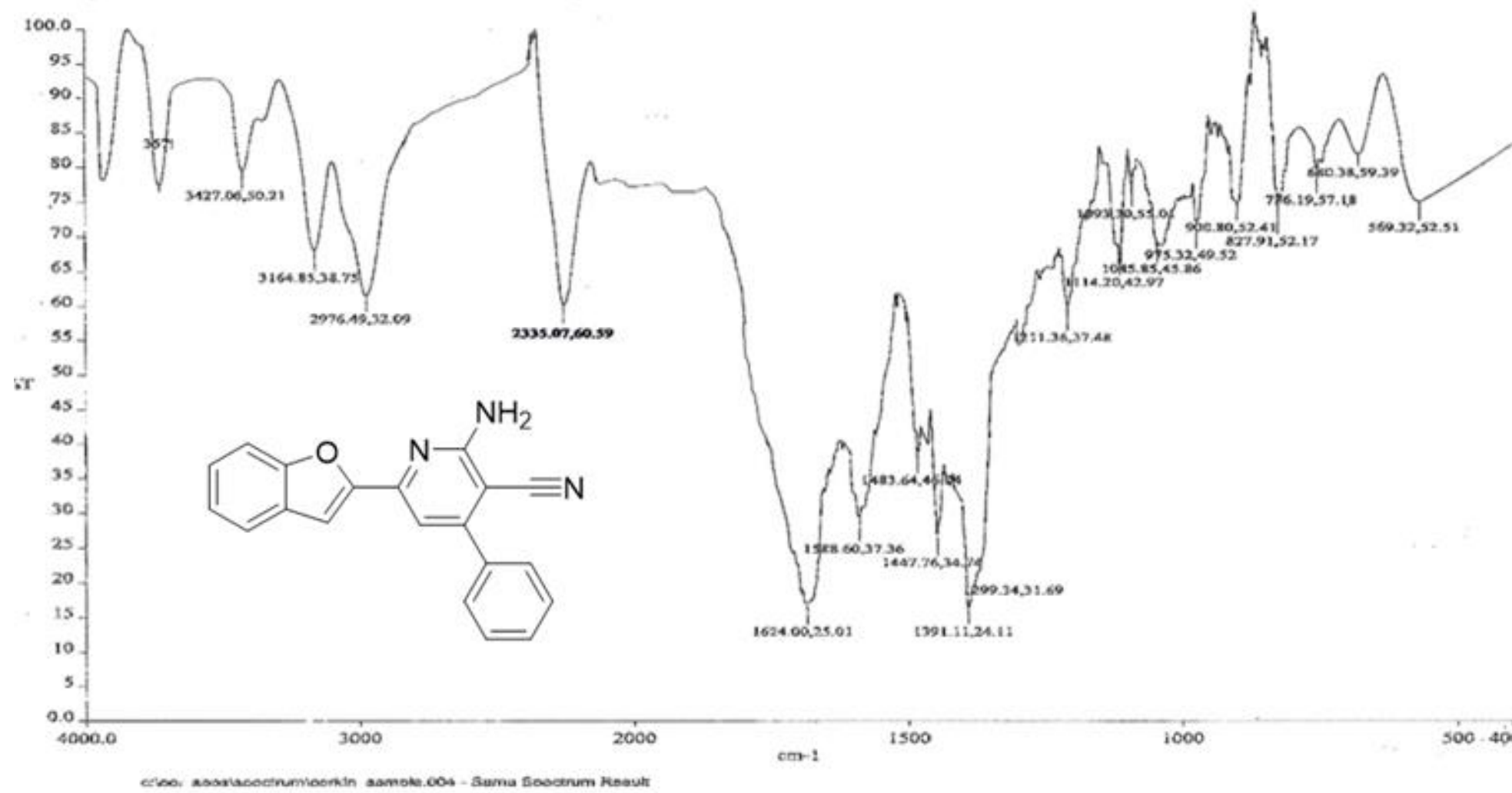

Fig S1: IR spectrum of 2-amino-6-(benzofuran-2-yl)-4-phenylnicotinonitrile (7a)

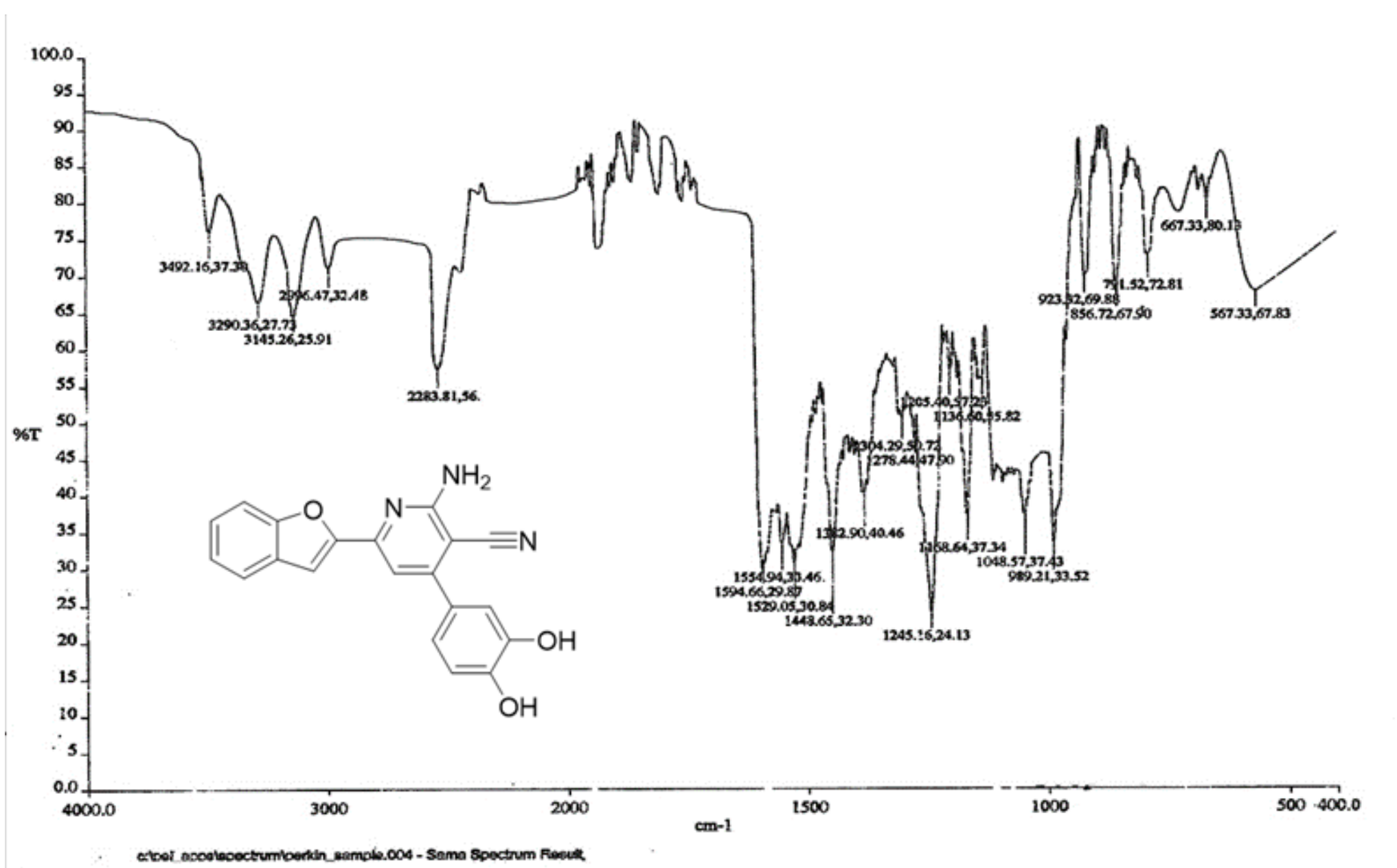

Fig S2: IR spectrum of 2-amino-6-(benzofuran-2-yl)-4-(3,4-dihydroxyphenyl) nicotinonitrile (7b)

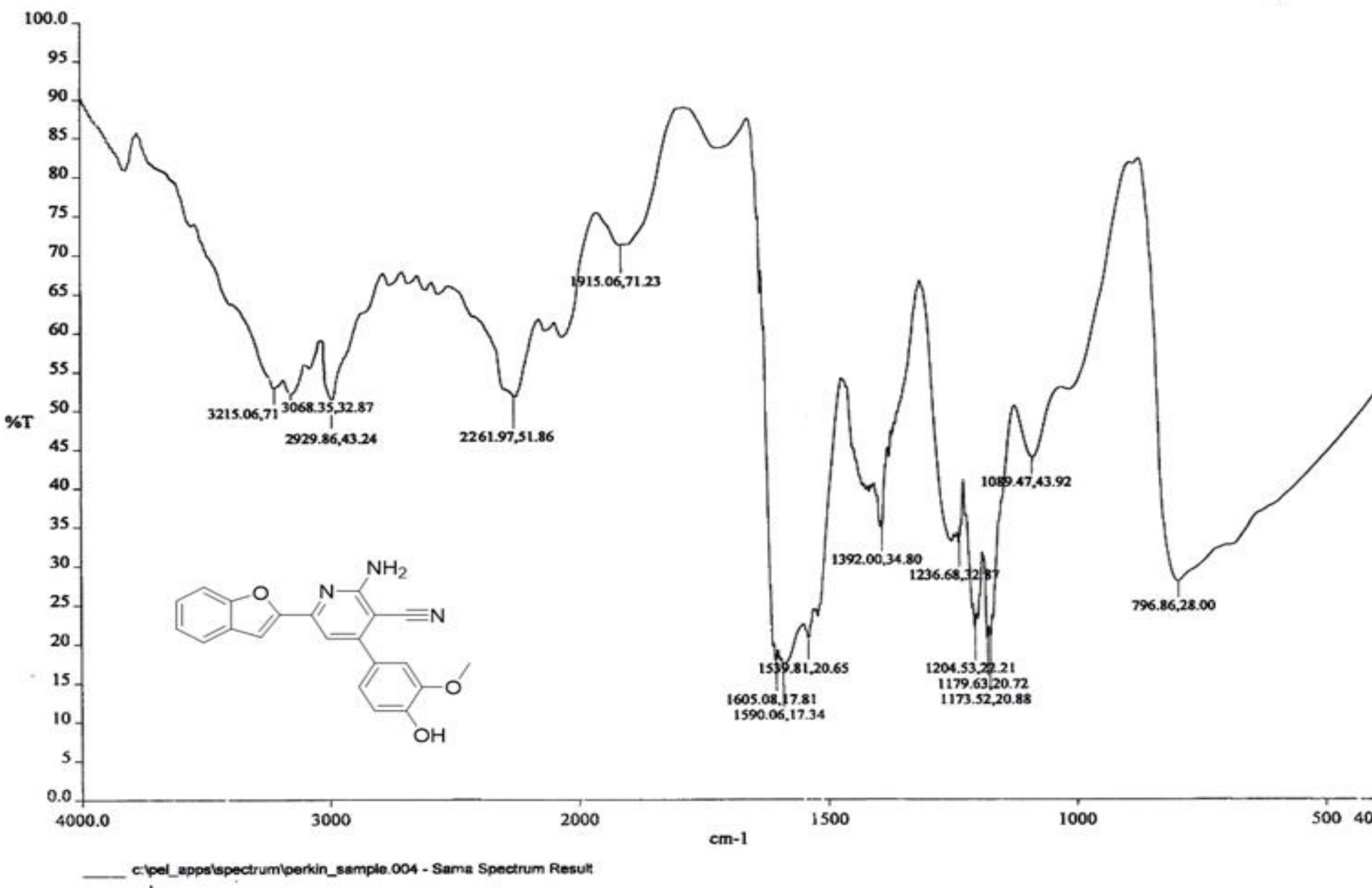

Fig S3: IR spectrum of 2-amino-6-(benzofuran-2-yl)-4-(4-hydroxy-3-methoxyphenyl) nicotinonitrile (7c)

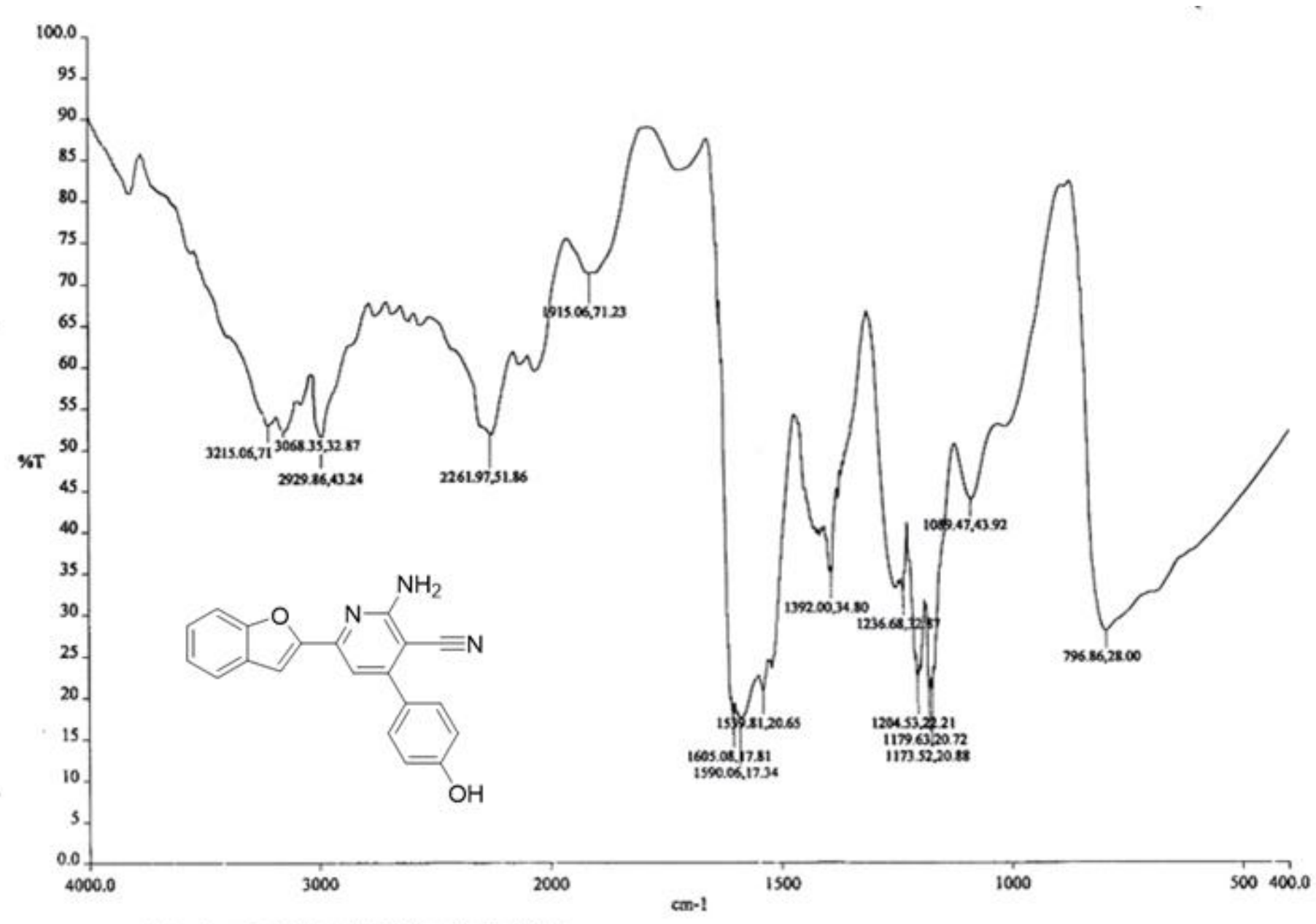

Fig S4: IR spectrum of 2-amino-6-(benzofuran-2-yl)-4-(4-hydroxyphenyl) nicotinonitrile (7d)

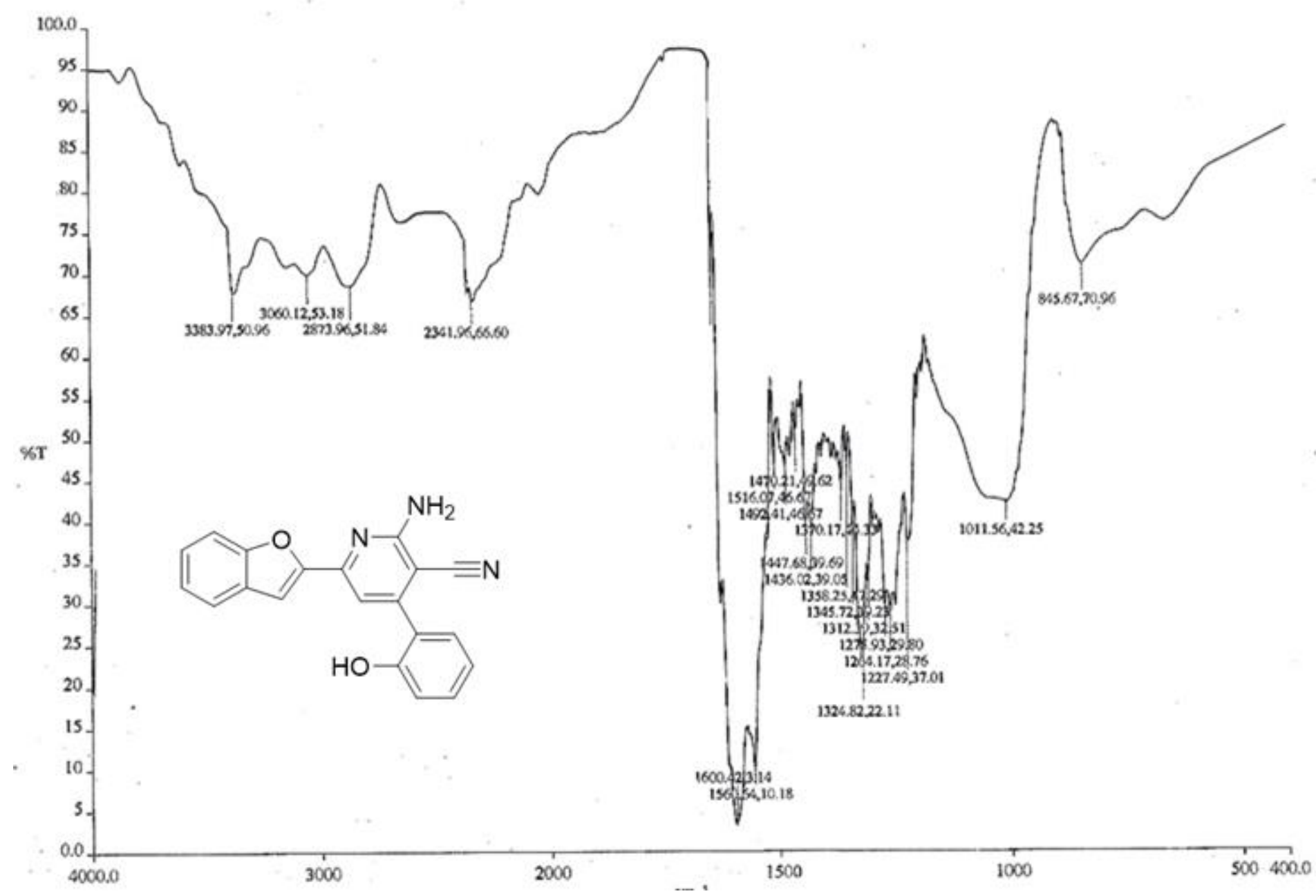

Fig S5: IR spectrum of 2-amino-6-(benzofuran-2-yl)-4-(2-hydroxyphenyle) nicotinonitrile (7e)

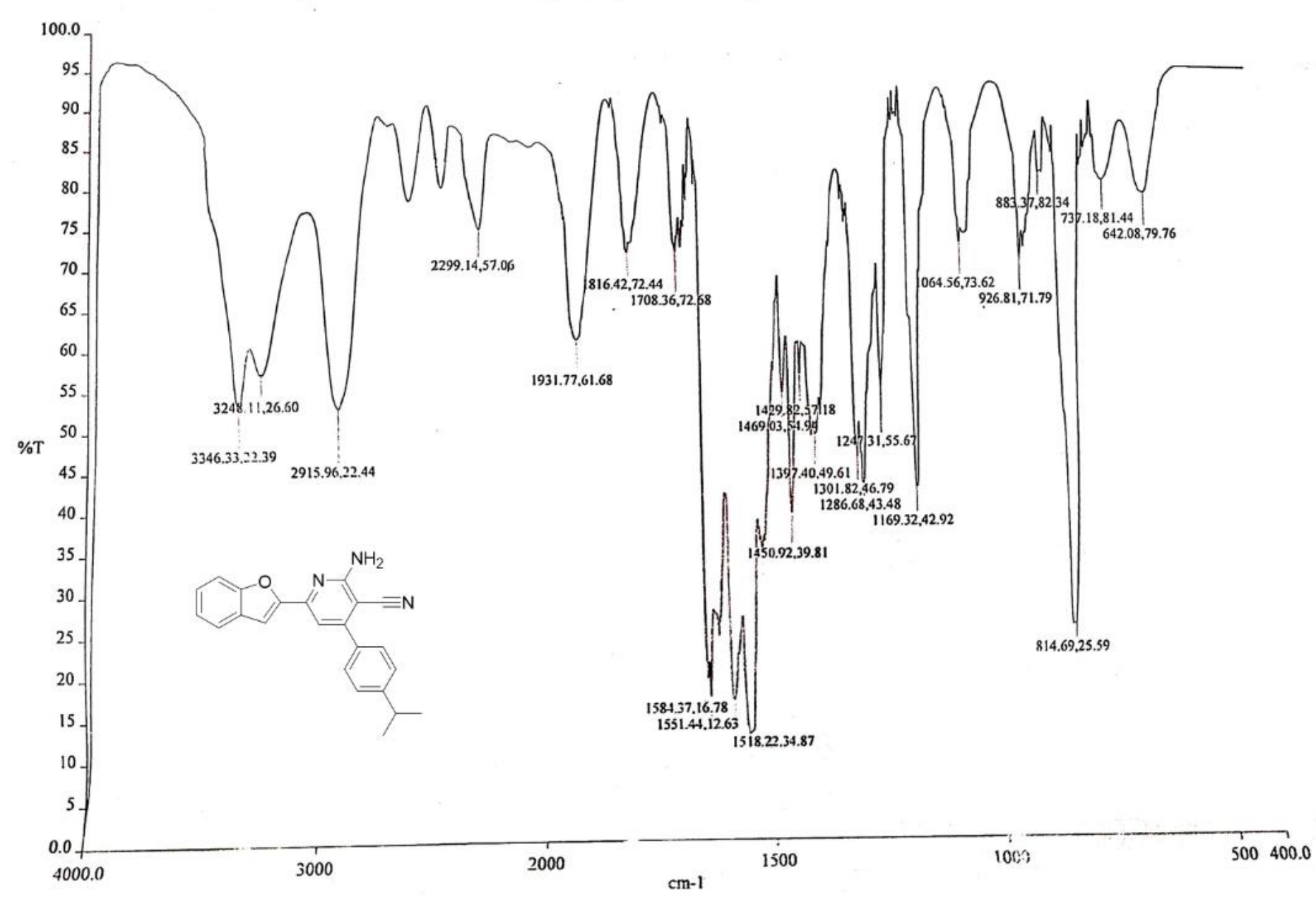

Fig S6: IR spectrum of 2-amino-6-(benzofuran-2-yl)-4-(4-isopropylphenyl) nicotinonitrile (7f)

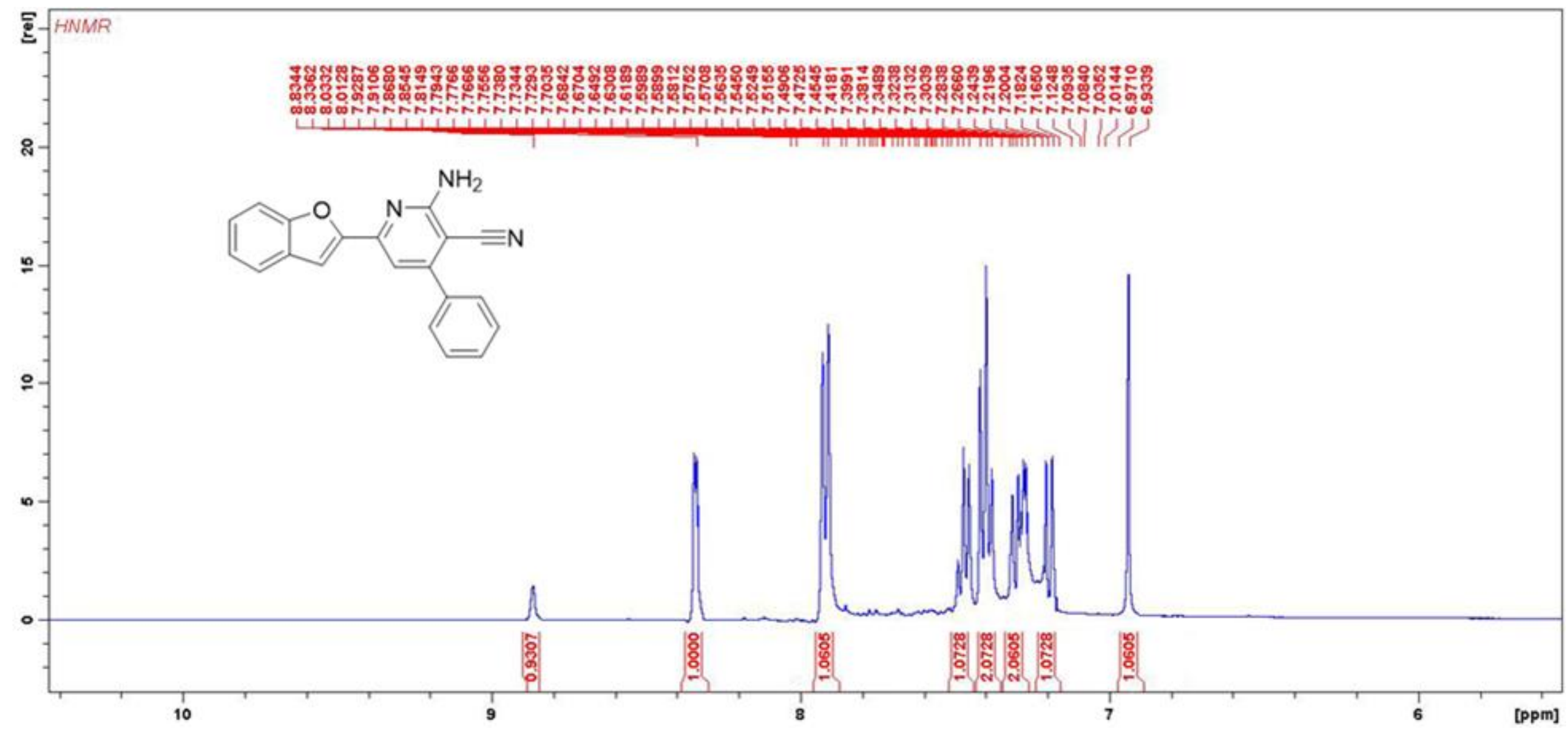

Fig S7: ^{1}HNMR spectrum of 2-amino-6-(benzofuran-2-yl)-4-phenylnicotinonitrile (7a)

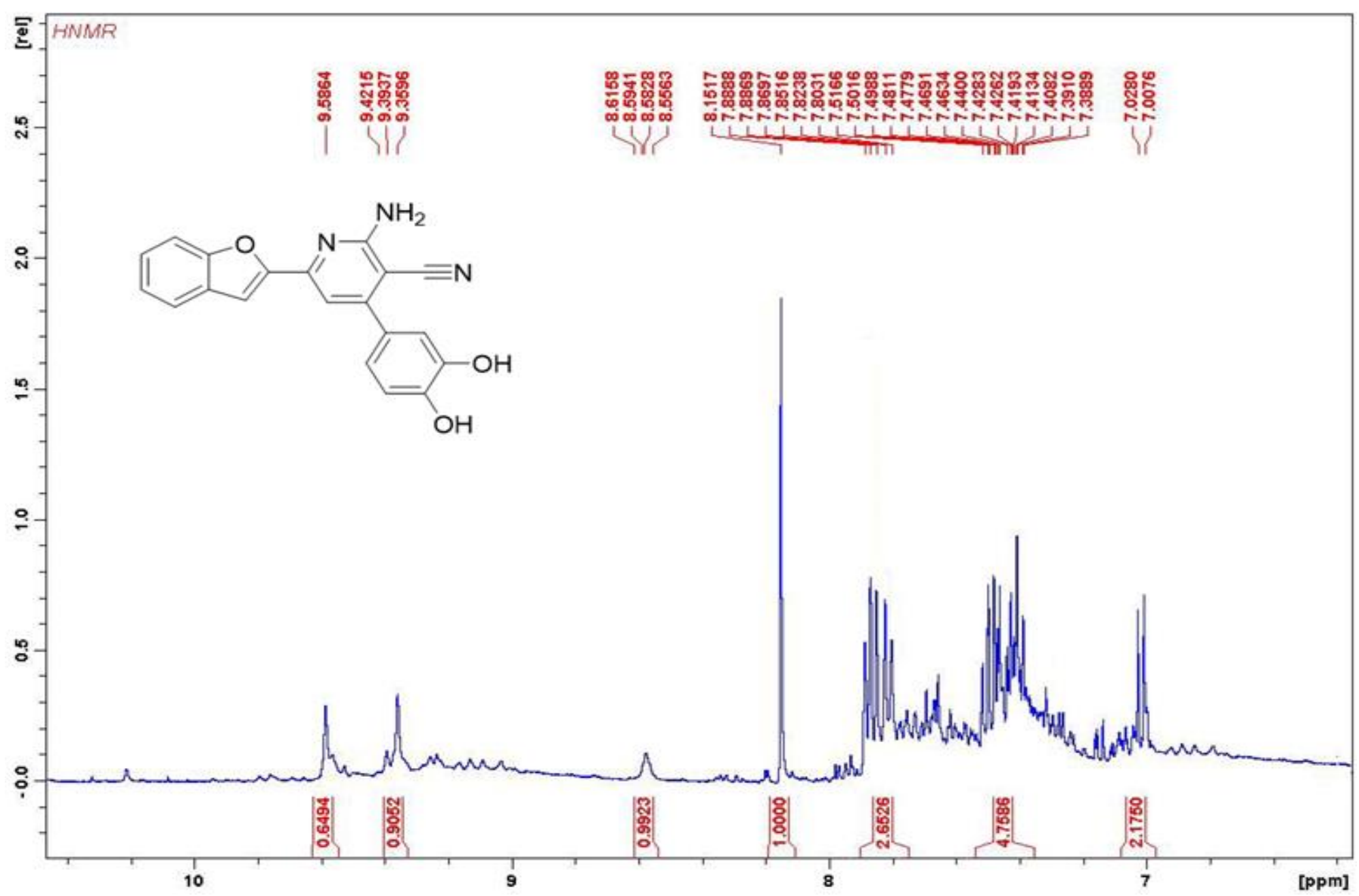

Fig S8: ^{1}HNMR spectrum of 2-amino-6-(benzofuran-2-yl)-4-(3,4-dihydroxyphenyl) nicotinonitrile (7b)

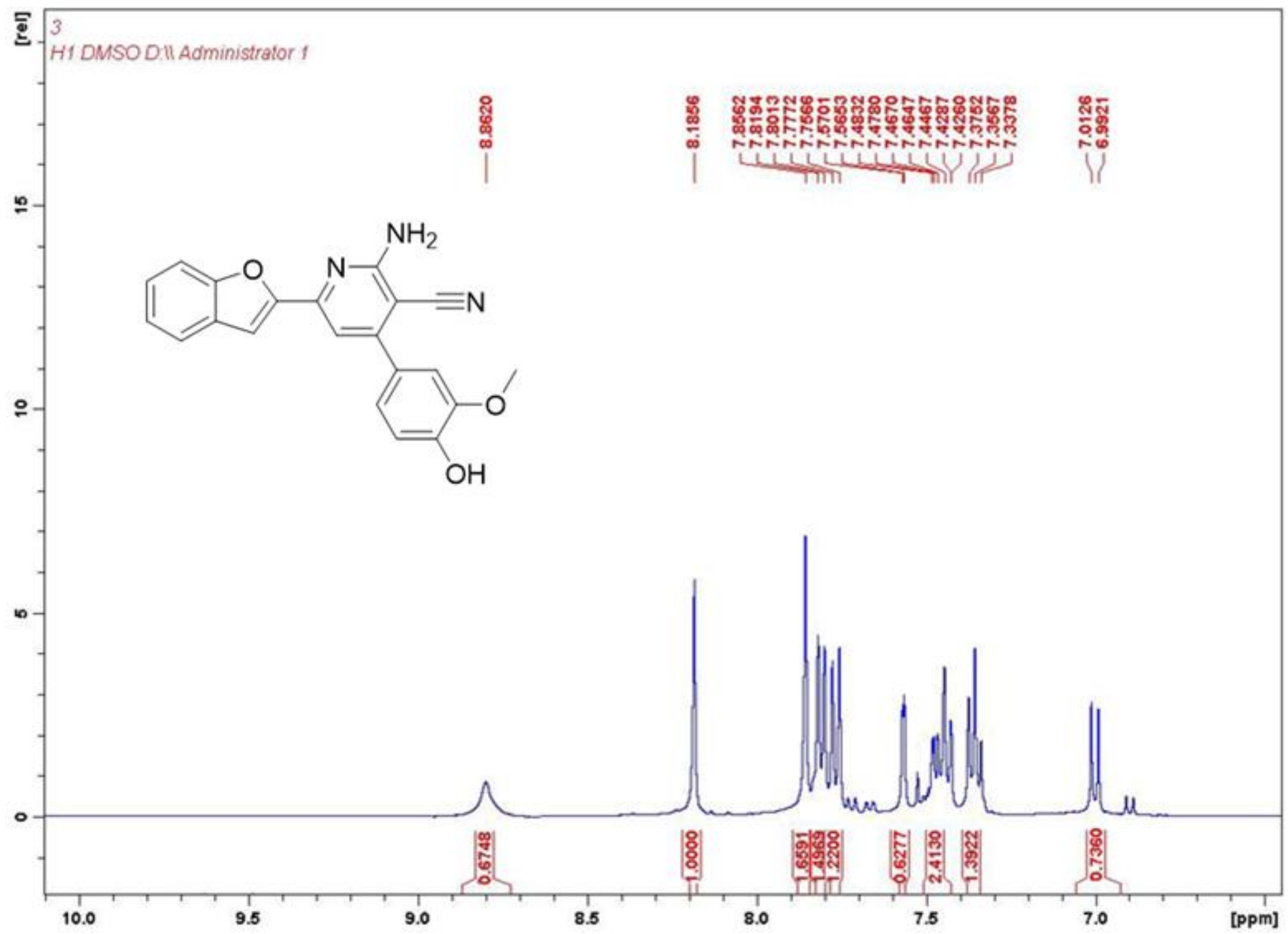

Fig S9: ^{1}HNMR spectrum of 2-amino-6-(benzofuran-2-yl)-4-(4-hydroxy-3-methoxyphenyl) nicotinonitrile (7c)

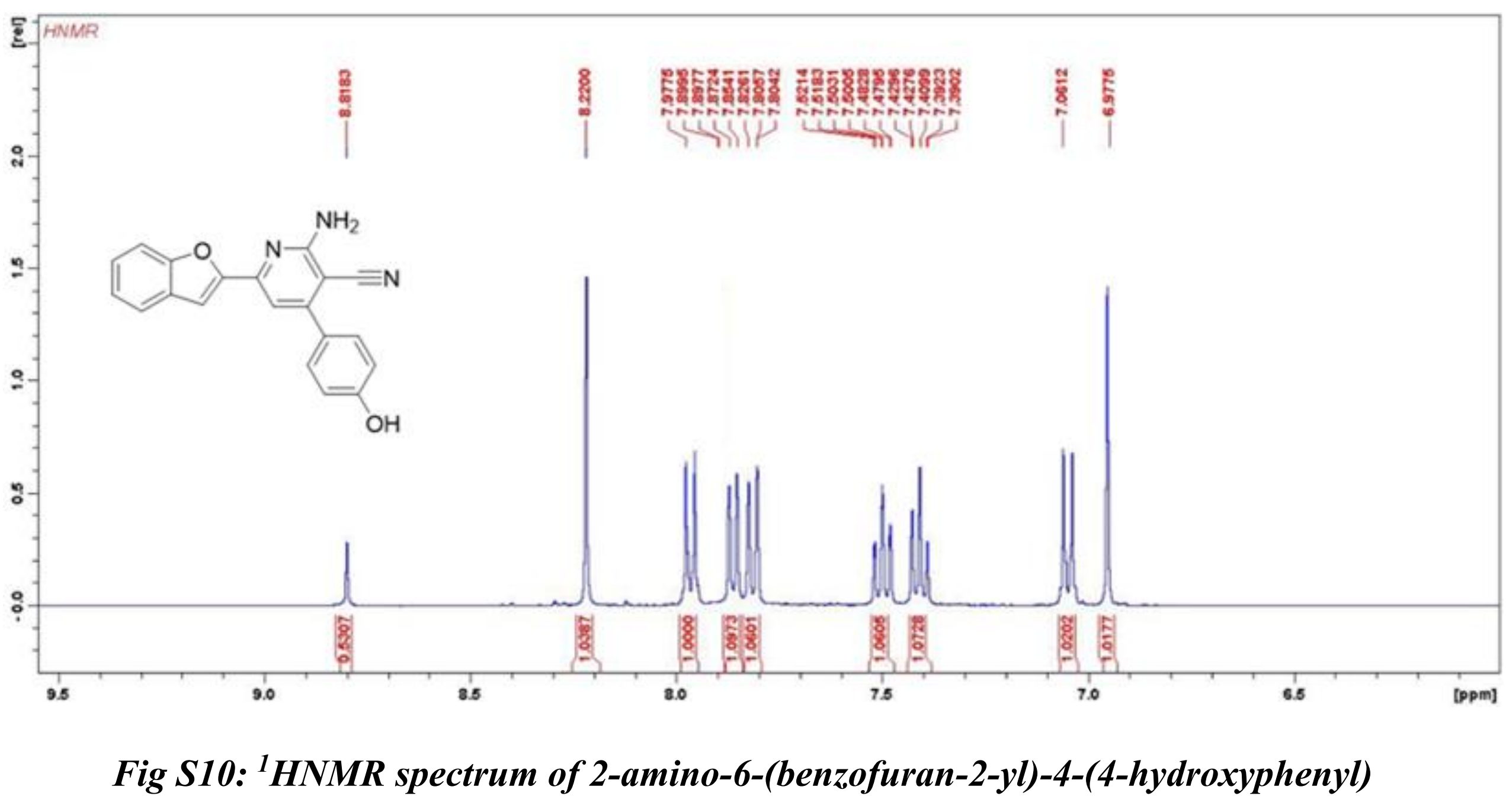

Fig S10: ^{1}HNMR spectrum of 2-amino-6-(benzofuran-2-yl)-4-(4-hydroxyphenyl) nicotinonitrile (7d)

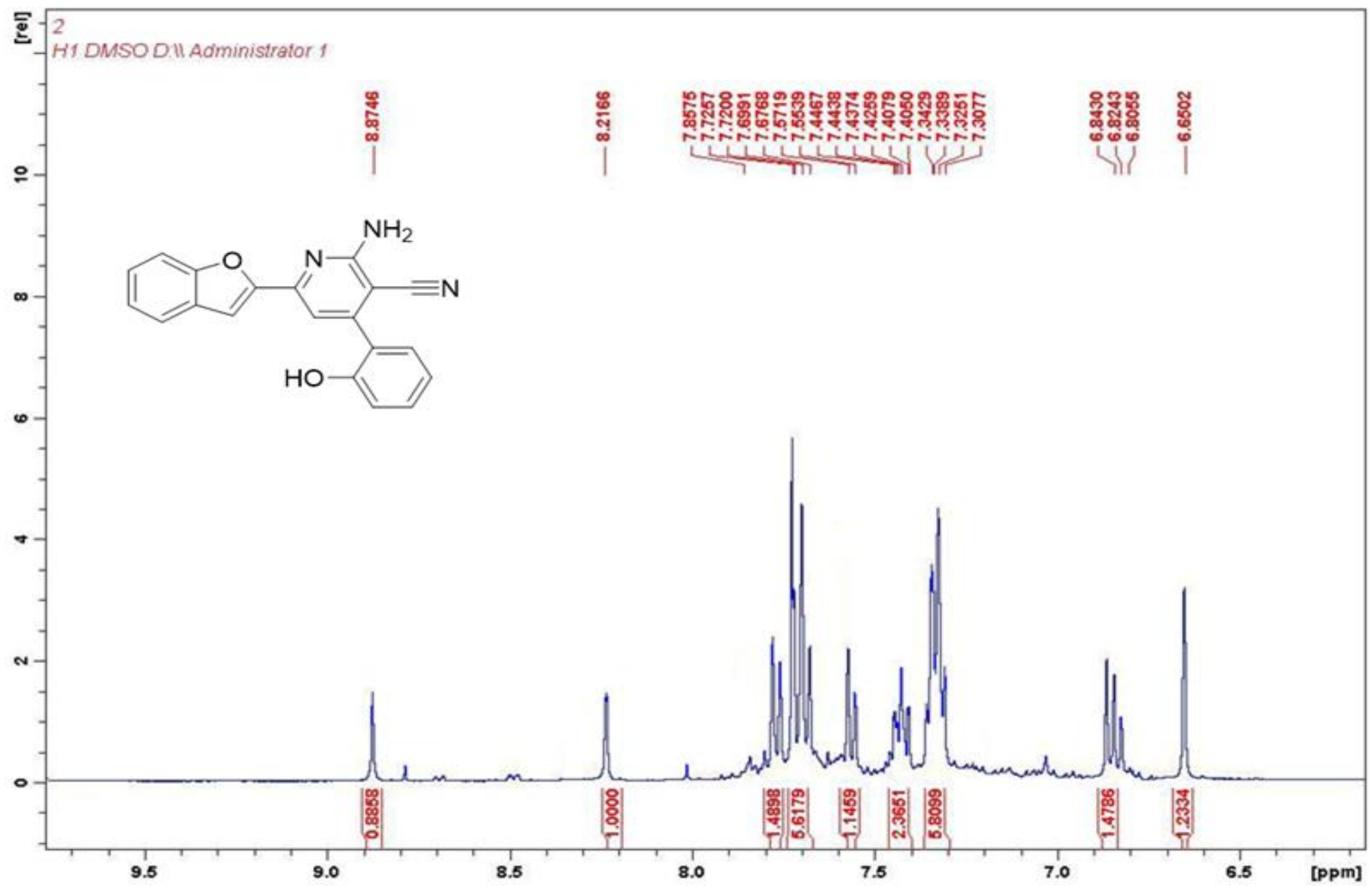

Fig S11: ^{1}HNMR spectrum of 2-amino-6-(benzofuran-2-yl)-4-(2-hydroxyphenyle) nicotinonitrile (7e)

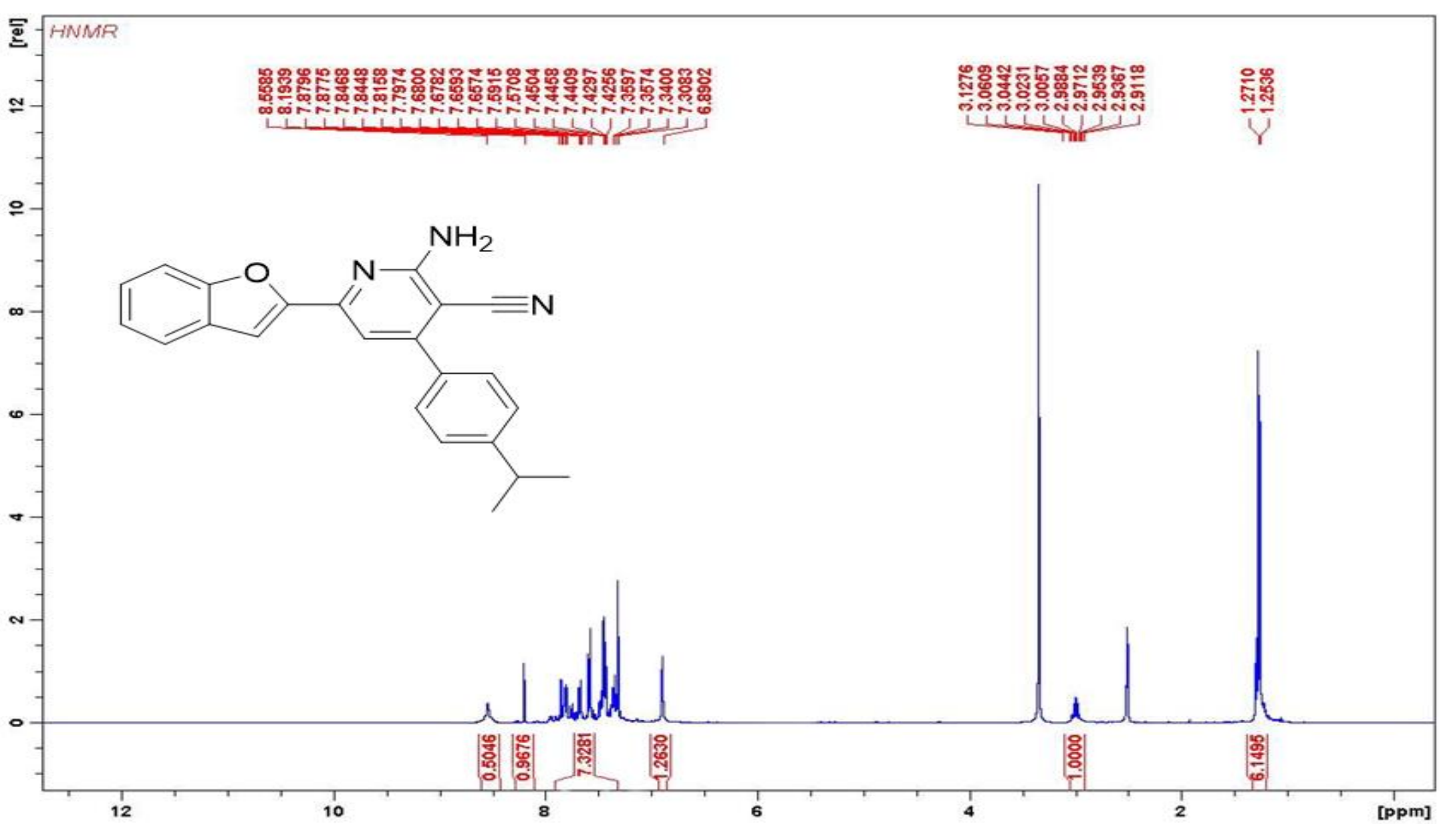

Fig S12: ^{1}HNMR spectrum of 2-amino-6-(benzofuran-2-yl)-4-(4-isopropylphenyl) nicotinonitrile (7f)

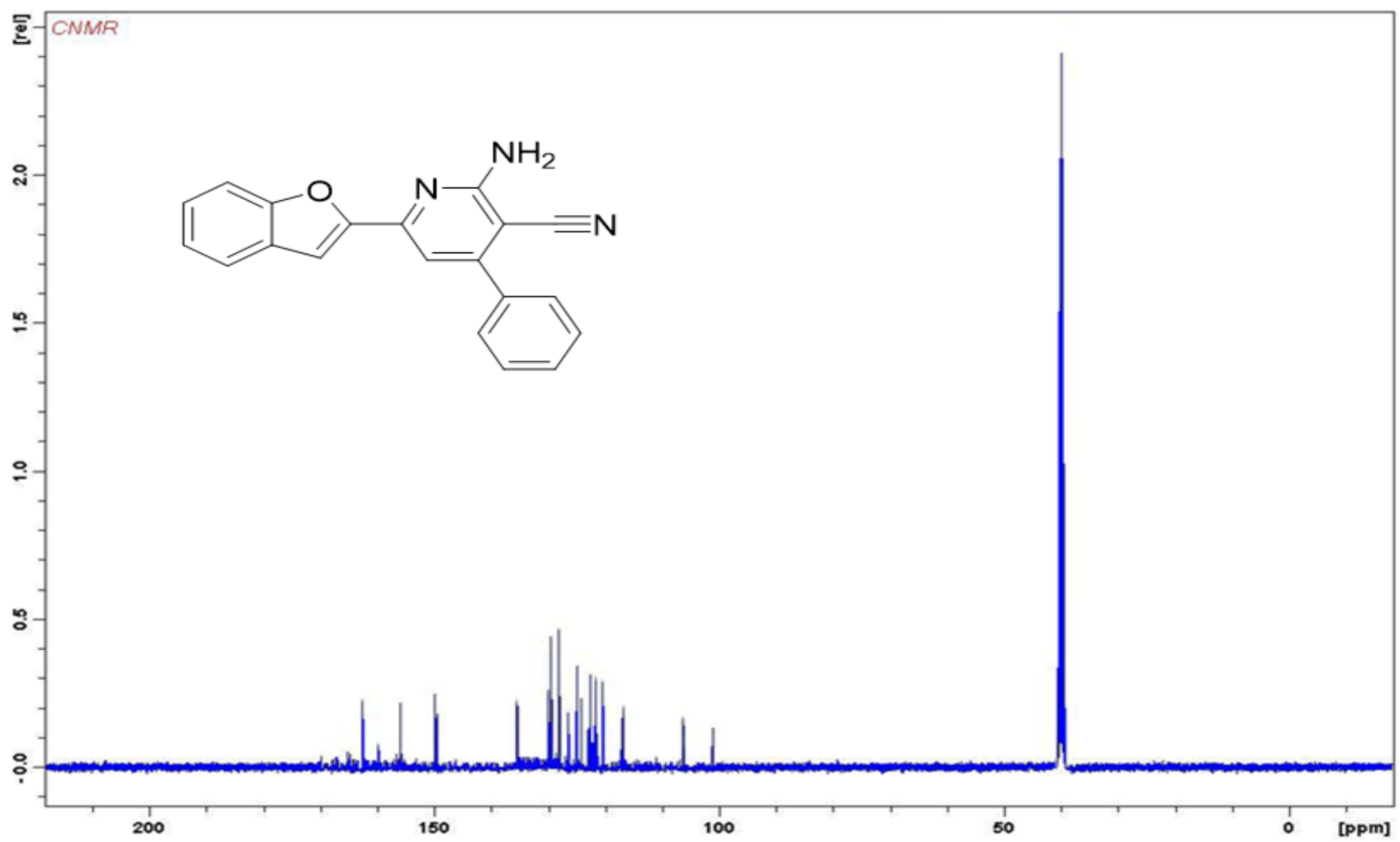

Fig S13: ^{13}CNMR spectrum of 2-amino-6-(benzofuran-2-yl)-4-phenylnicotinonitrile (7a)

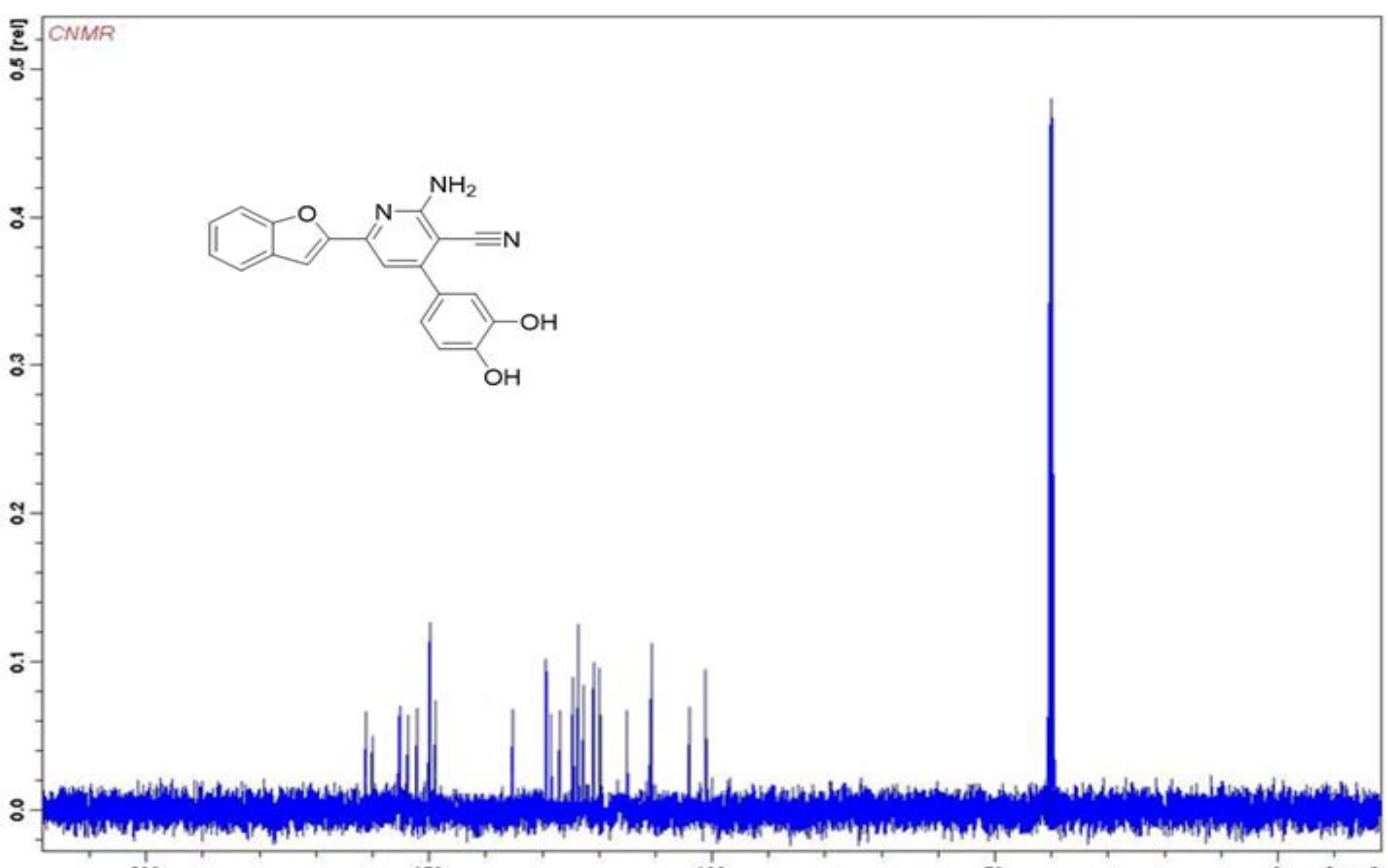

Fig S14: ^{13}CNMR spectrum of 2-amino-6-(benzofuran-2-yl)-4-(3,4-dihydroxyphenyl) nicotinonitrile (7b)

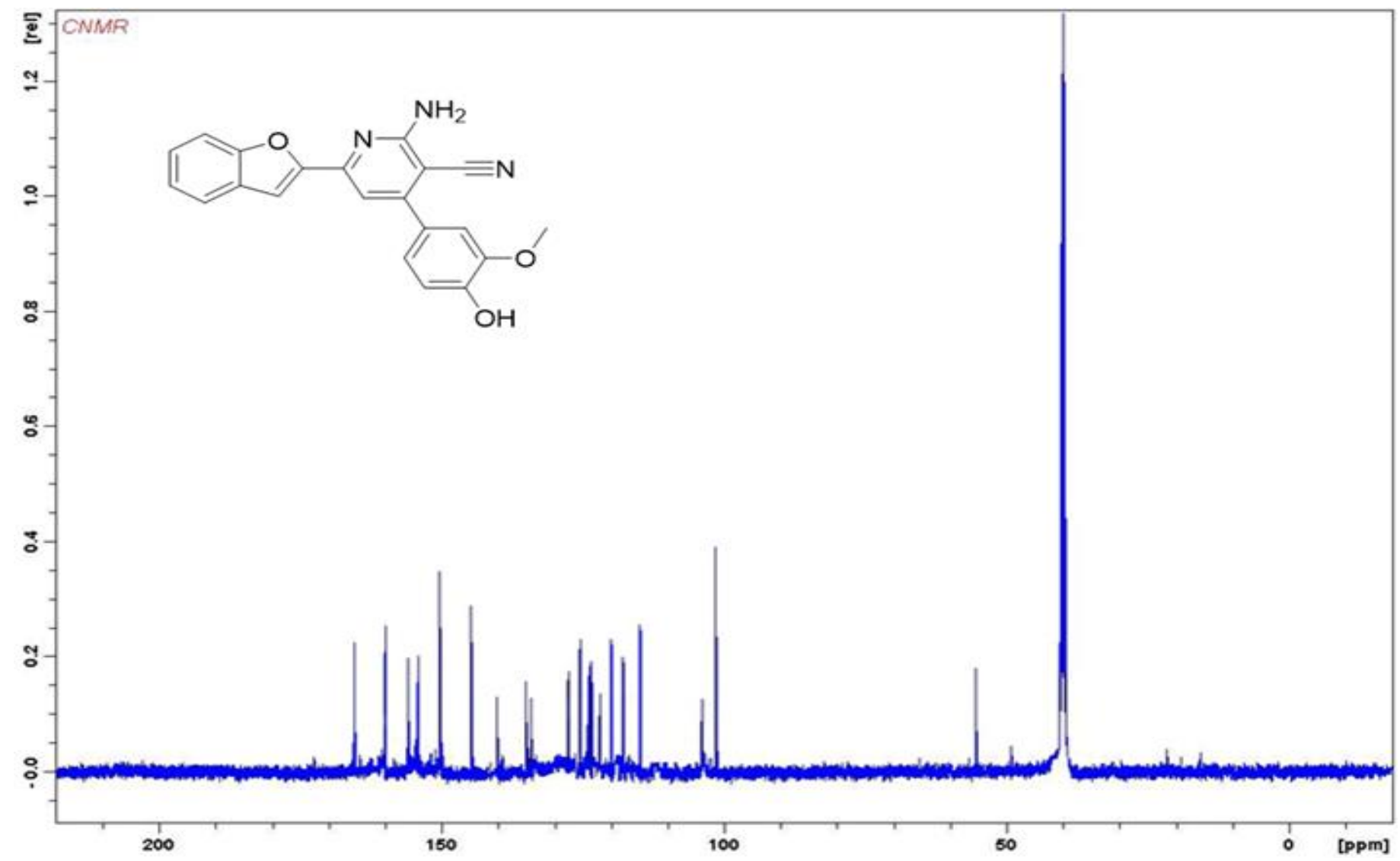

Fig S15: ^{13}CNMR spectrum of 2-amino-6-(benzofuran-2-yl)-4-(4-hydroxy-3-methoxyphenyl) nicotinonitrile (7c)

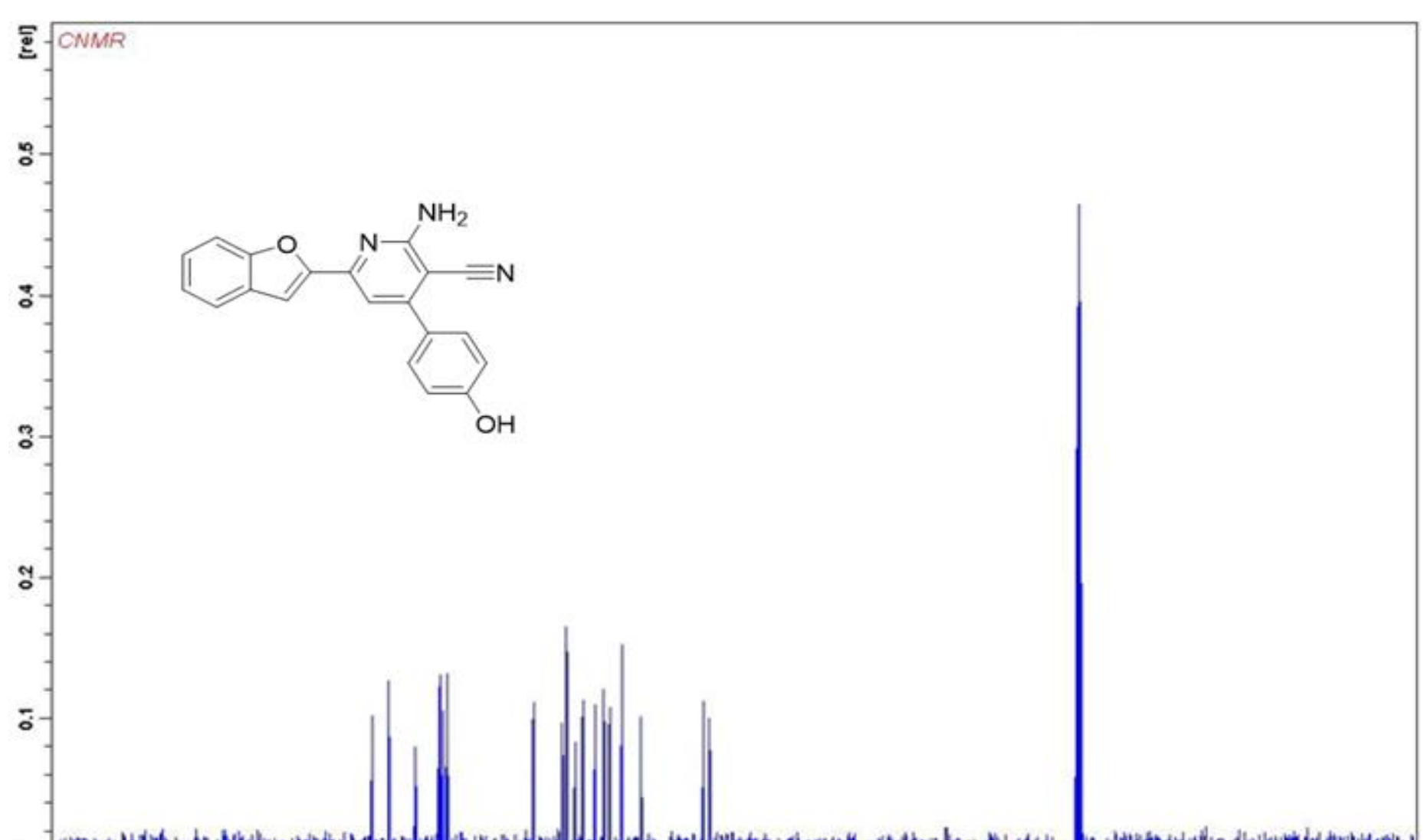

Fig S16: ^{13}CNMR spectrum of 2-amino-6-(benzofuran-2-yl)-4-(4-hydroxyphenyl) nicotinonitrile (7d)

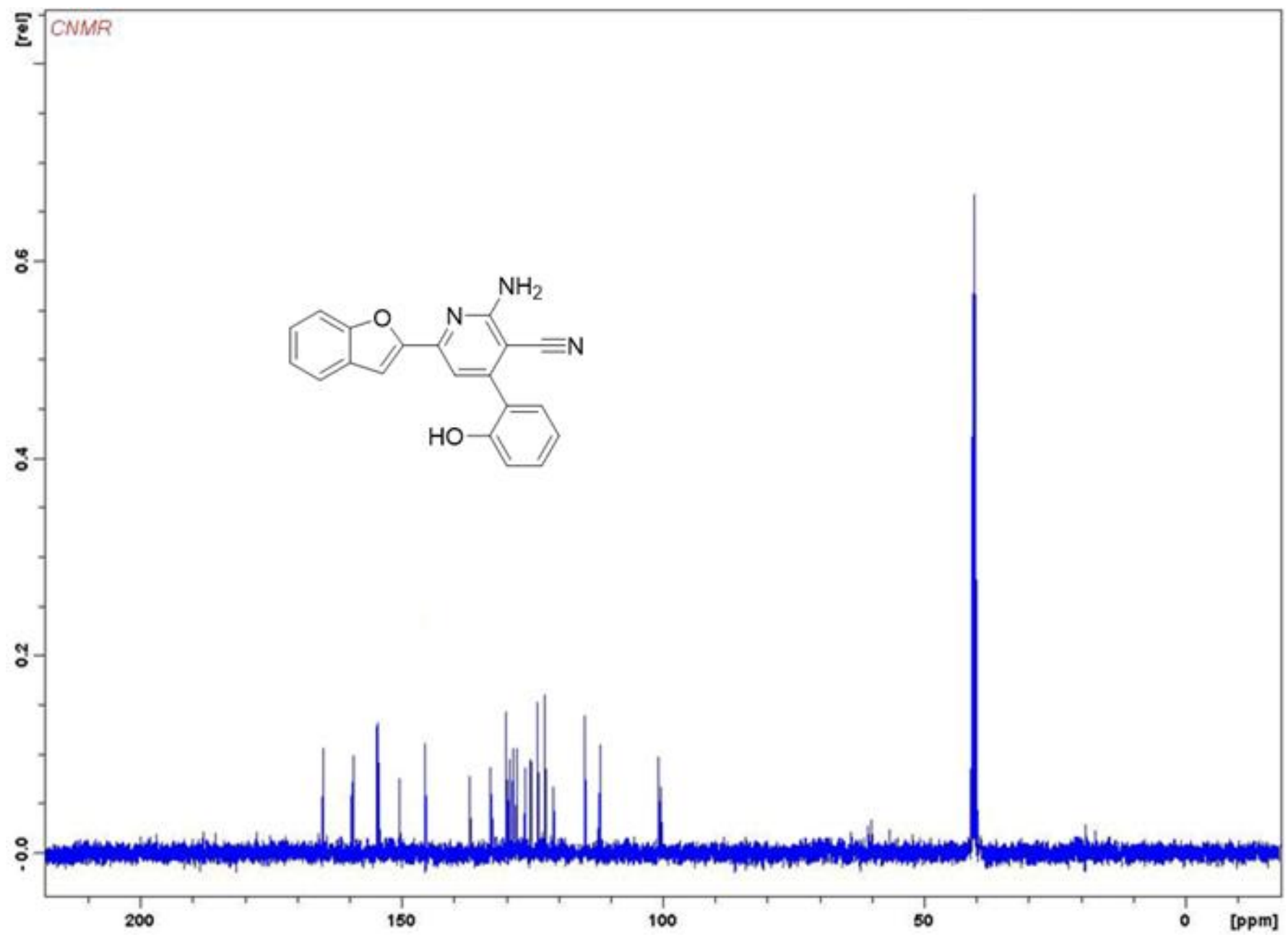

Fig S17: ^{13}CNMR spectrum of 2-amino-6-(benzofuran-2-yl)-4-(2-hydroxyphenyle) nicotinonitrile (7e)

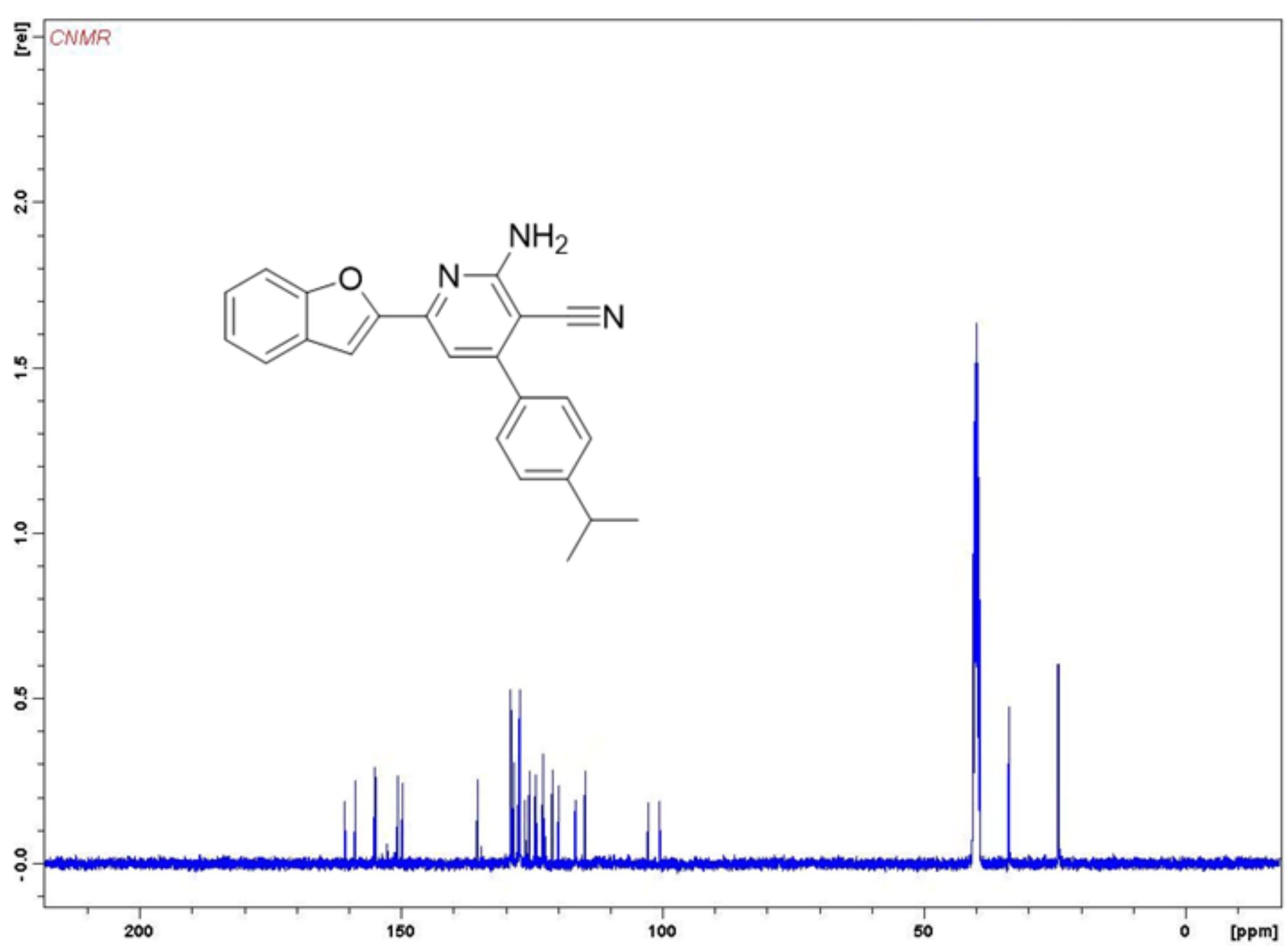

Fig S18: ^{13}CNMR spectrum of 2-amino-6-(benzofuran-2-yl)-4-(4-isopropylphenyl) nicotinonitrile (7f)
